# *RAB5B*: explore its carcinogenic potential and understand its role, prognostic value and biological significance in pan-cancer

**DOI:** 10.3389/fimmu.2025.1619733

**Published:** 2025-08-06

**Authors:** Jing Zhu, Chun Yu, Xin Xu, Chu Zou, Jiaqi Li, Rui Wang, Xinchao Li, Aijun Sun, Shiyan Wang, Chao Jiang

**Affiliations:** ^1^ Department of Oncology, The Affiliated Huaian No.1 People’s Hospital of Nanjing Medical University, Huai’an, China; ^2^ General Surgery Department, Lianshui People’s Hospital Affiliated to Kangda College of Nanjing Medical University, Huai’an, Jiangsu, China; ^3^ Department of Thyroid and Breast Oncological Surgery,Huai’an Second People’s Hospital, TheAffiliated Huaian Hospital of Xuzhou Medical University, Huai’an, Jiangsu, China; ^4^ Faculty of Life Science and Food Engineering, Huaiyin Institute of Technology, Huaian, Jiangsu, China

**Keywords:** RAB5B, pan-cancer, tumor immunity, prognosis, drug sensitivity of immunotherapy

## Abstract

**Background:**

The *RAB5B* protein belongs to the *RAB* family and is primarily localized to early endosomes. It regulates the endocytic pathway through its GTPase activity, thereby affecting various aspects such as cell signaling and metabolic regulation. Dysfunction of *RAB5B* is closely associated with the progression and deterioration of multiple types of tumors. Although studies have revealed the functional mechanisms of *RAB5B* in specific tumor types, its role in pan-cancer and the underlying molecular mechanisms still lack in-depth analysis.

**Methods:**

A comparative analysis of *RAB5B* gene expression was conducted using transcriptomic datasets from Cancer Genome Map (TCGA) and Genotype-Tissue Expression (GTEx), followed by tissue distribution profiling via Human Protein Atlas (HPA) and GeneMANIA to map its expression across human tissues. The TISCH database identified primary cell types expressing *RAB5B* within the tumor microenvironment, while univariate Cox regression modeling evaluated its prognostic significance in cancer outcomes. Integrative genomic analyses using cBioPortal and Gene Set Cancer Analysis (GSCA) further characterized *RAB5B*’s genomic alterations and cancer-specific profiles. Gene_DE module 2.0 (TIMER 2.0) deciphered associations between *RAB5B* expression and tumor-infiltrating immune cell subsets. To elucidate functional mechanisms, Gene Set Enrichment Analysis (GSEA) and Gene Set Variation Analysis (GSVA) identified biological pathways co-regulated with *RAB5B*, and in silico approaches combining CellMiner with molecular docking predicted interactions between *RAB5B* and anticancer drugs. *In vitro* wound healing assays were performed to validate *RAB5B*’s role in modulating cellular migration dynamics, complementing bioinformatics findings with experimental evidence.

**Results:**

*RAB5B* protein expression varied significantly across tumors, with different prognostic values. In most malignancies, *RAB5B* expression correlated positively with Copy Number Variation (CNV) and methylation. It also correlated significantly with immunotherapy biomarkers and responses. ESTIMATE and immune infiltration analyses highlighted *RAB5B*’s link to immunosuppression, emphasizing its role in immune regulation. Molecular docking and experimental validation showed that downregulating *RAB5B* inhibited cell proliferation and reduced cancer cell migration.

**Conclusion:**

Our study revealed the key role of *RAB5B* in tumor biomarkers. *RAB5B* inhibits ectopic metastasis of tumor cells mainly by regulating the process of cell adhesion and migration. This discovery is of great significance for developing new anticancer inhibitors.

## 1 Introduction

As a major culprit endangering human health, the incidence and death cases of cancer are rising year by year, which has constituted a severe challenge in the field of global public health (1). The complex development process of cancer involves multiple genes, highlighting the urgency of exploring new diagnostic markers and treatment options. Therefore, conducting a comprehensive analysis of cancer expression profiles to reveal their correlations with clinical prognosis and underlying molecular mechanisms is crucial.The rapid advancement of modern sequencing technologies and bioinformatics has enabled us to gain deeper insights into the intricate molecular mechanisms of cancer. Bioinformatics analysis has driven the transformation of cancer research from traditional pathology-based understanding to molecular-based precise diagnosis, allowing cancer classification systems to more accurately reflect therapeutic responses. In recent years, a comprehensive database like the Cancer Genome Map (TCGA) has collected huge tumor genome and clinical data sets (2), providing a vital information treasure house for uncovering the complicated mechanism of cancer. By integrating multi-omics data, we can comprehensively analyze gene expression across various cancer types and delve into the complex relationships among gene expression characteristics, clinical prognostic indicators, and signal transduction pathways. With the widespread application of these technologies, tumor bioinformatics analysis is ushering in a new era of cancer research.

*RAB* protein family is a kind of GTP binding protein widely existing in eukaryotic cells, which belongs to Ras superfamily and has GTP enzyme activity (3). They play vital roles in biological processes such as cell membrane transport and vesicle transport, with each member exhibiting unique functions. For instance, *RAB7* is involved in the membrane fusion of late endosomes with lysosomes and protein transport to lysosomes; *RAB10* participates in membrane transport, substance metabolism, and is associated with tumor occurrence and development (4). *RAB10* participates in membrane transport, substance metabolism, and is associated with tumor occurrence and development (5). *RAB5B* plays a key role in endocytosis and regulation of endocytosis pathway. As a molecular switch, it accurately regulates the formation, transportation, binding and fusion of vesicles thus ensuring the smooth transport of substances and signal transduction in cells (6). Notably, *RAB5B* is also involved in regulating the metastasis and invasion of cancer cells (7). *RAB5B* can change the migration and adhesion characteristics of cancer cells by affecting vesicle transport and signal transduction in cells, thus promoting the spread and metastasis of cancer cells and increasing the malignant degree and prognosis risk of cancer. However, current research on *RAB5B* predominantly focuses on individual cancer types, and its comprehensive roles and therapeutic relevance across diverse malignancies remain to be further explored.

Delving into gene functions is pivotal in biomedical exploration. *RAB5B* has garnered significant attention due to its core role in intracellular material transport and membrane vesicle fusion. Within the *RAB*5 subfamily, *RAB5B* coordinates with other members in various stages of endocytosis, ensuring accurate and efficient fusion of endocytic vesicles with early endosomes for precise sorting and efficient transport of substances. The GTP activity of *RAB5B* acts as a “switch” in this process, regulating vesicle fusion (8). The regulatory effect of *RAB5B* is not limited to normal cell physiological activities. Studies have shown that in many cancers, the abnormal expression and function of *RAB5B* are closely related to the progress of the disease. In some tumor cells, the abnormal expression of *RAB5B* may disturb the intracellular material transport system, and then have a far-reaching impact on the malignant behaviors such as cell proliferation, apoptosis, invasion and migration. *RAB5B* is regarded as a potential therapeutic target in cancer research. By precisely regulating the expression or function of *RAB5B*, it is anticipated that the substance transport chain of cancer cells can be interrupted, thereby inhibiting their malignant behaviors such as proliferation, invasion, and metastasis. For instance, targeting *RAB5B* through small molecule inhibitors or gene editing technology offers a means to further explore its potential applications in cancer therapy. At the same time, the in-depth study of the complex interaction between *RAB5B* and other signal pathways will also help us to reveal the mystery of cancer occurrence and development more comprehensively and open up new ideas and ways for the development of precision medicine (9). As a key regulator of intracellular substance transport and vesicle fusion the in-depth exploration of its function will not only provide us with strong theoretical support and practical basis for revealing the pathogenesis of diseases (10, 11), but also open up broad prospects for us to find new therapeutic targets.

This study aims to comprehensively review the research progress of *RAB5B* in different types of tumors. Leveraging authoritative databases such as TCGA, Human Protein Atlas (HPA), Cancer Cell Line Encyclopedia (CCLE), Genotype-Tissue Expression (GTEx), and Tumor Immune Single-cell Hub (TISCH), we have conducted detailed studies on the expression levels of *RAB5B* in various malignant tumors and its potential impact on patient prognosis. Furthermore, we have performed genome-wide variation analysis and cancer prognosis evaluation to explore the intrinsic relationships between *RAB5B* and key factors such as DNA methylation, CNV, tumor mutation burden (TMB), microsatellite instability (MSI), and immune infiltration. We have also investigated the potential association between *RAB5B* and immune checkpoint inhibitory therapy.

To gain a more comprehensive understanding of *RAB5B*’s function in 33 diverse cancers, multidimensional analysis was conducted, encompassing the co-expression of *RAB5B* with matrix score, immune score, ESTIMATE score, MMR (Mismatch Repair) status, and immune-related genes. Additionally, we performed docking experiments between drug candidates and their target molecules and sensitivity tests on *RAB5B* using Gene Set Enrichment Analysis (GSEA), Gene Set Variation Analysis (GSVA), and various anticancer drugs. Despite achieving these preliminary research outcomes, many unknowns regarding the specific molecular mechanism of *RAB5B* in other cancers remain to be further explored. Only through a deep understanding of these mechanisms can more precise and effective drug treatment strategies be developed, ultimately bringing better treatment prospects to cancer patients. This study focuses on the role of *RAB5B* gene in the function of breast cancer cells, especially its influence on cell viability and migration potential. The *RAB5B* gene was successfully knocked out in breast cancer cell lines using siRNA technology, and the corresponding cell deletion model was constructed. The results of cell activity test clearly showed that the knock-down of *RAB5B* gene significantly inhibited the survival rate of breast adenoma cells. In addition, through scratch test and migration experiment, it was found that the migration ability of cells in *RAB5B* knock-down group was significantly lower than that in control group, and its migration distance was also significantly shortened. These findings underscore the core role of *RAB5B* in tumor development and progression, offering new perspectives for breast cancer treatment strategies and potential targets. Furthermore, they reinforce the position of *RAB5B* as a reliable indicator for prognosis evaluation in various cancers. Additionally, its role in tumor immunity, affecting immune cell infiltration, TMB, and MSI, as well as its correlation with breast cancer drugs, suggests its potential as a biomarker for breast cancer immunotherapy, laying a solid theoretical foundation for further exploring tumor immunity mechanisms in breast cancer.

## 2 Method

### 2.1 Open data and analysis methods

Bubble charts illustrating various diseases or phenotypes associated with *RAB5B* protein were presented with the assistance of the Open Targets platform (URL: https://platform.opentargets.org/). To determine the specific location of *RAB5B* protein within cells, information from the HPA database (URL: https://www.proteinatlas.org/) was referenced, confirming the protein’s detailed subcellular location (12). Additionally, RNA-seq data in units of transcripts per million (TPM) were obtained from the UCSC XENA database encompassing 33 types of cancer tissues and their corresponding normal tissues (13). For data consistency, the Toil pipeline was employed to uniformly preprocess these data. Subsequently, the Gene_DE module 2.0 (TIMER2.0) was utilized to thoroughly analyze differences in *RAB5B* mRNA expression between cancer tissues and adjacent normal tissues (14). Furthermore, data from the CCLE database (URL: https://portals.broadinstitute.org/ccle/) were acquired to evaluate *RAB5B* expression levels across 29 distinct tissues. Except for the global differential gene expression analysis of cancer, all the TPM-formatted mRNA expression data and related clinical data relied on by the follow-up analysis were obtained from the TCGA cancer global cohort data set downloaded from the GDC data portal (2).

The GeneMANIA platform (http://www.genemania.org) was utilized to construct a protein-protein interaction (PPI) network (15), with the objective of obtaining interaction information relevant to *RAB5B* protein. This analytical step was critical for predicting *RAB5B* gene function, formulating functional hypotheses, and identifying genes with analogous biological roles. Through PPI network characterization, the study dissected *RAB5B* features across multiple dimensions, including physical interaction patterns, co-expression profiles, predictive network relationships, co-localization phenomena, genetic interactions, pathway participation, and shared protein domain architectures.

### 2.2 Single cell analysis and spatial resolution transcriptome

When the TISCH network tool (http://tisch1.comp-genomics.org/) was used to perform single cell data analysis, the following parameters were configured: gene *RAB5B*, major cell lines, and all types of cancers. Subsequently, the expression level of *RAB5B* in various cell types was evaluated and displayed in the form of a thermogram (16). Additionally, upon utilizing the SpatialDB online tool (https://www.spatialomics.org/SpatialDB/) (17), it was observed that the markers PECAM1 and VWF of M2 macrophages overlapped with the markers CD68 and CD163 of endothelial cells in skin melanoma.

### 2.3 Survival and prognosis analysis

Cox regression analysis was conducted with the assistance of the PanCanSurvPlot network platform (https://smuonco.shinyapps.io/PanCanSurvPlot/) to explore the correlation between *RAB5B* gene expression levels and multiple patient survival indices, including overall survival (OS), disease-specific survival (DSS), disease-free interval (DFI), and progression-free survival (PFS). This analysis was based on data from the TCGA database, with data specifically selected from the IlluminaHiSeq sequencing platform. To effectively evaluate the prognostic value of *RAB5B*, an optimized cutoff grouping strategy was adopted for each cancer type, where the cutoff was determined based on the specific clinical and biological characteristics of each cancer using an optimization algorithm to select the expression level that best distinguishes prognosis risk. Ultimately, the ‘forestplot’ package in R was utilized to present the analysis results, specifically in the form of risk ratio (HR) and its 95% confidence interval (95% CI), thereby visually displaying the statistical estimation of *RAB5B* expression’s impact on patient survival.

### 2.4 Summary of cancer-related genome tampering and *RAB5B* mutation

To gain a deeper understanding of the frequency of four key types of genomic variation in tumors—mutation, amplification, deep deletion, and multiple changes—the cancer type overview function of the cBioPortal platform (https://www.cbioportal.org/) was utilized for analysis (17). Concurrently, the Gene Set Cancer Analysis (GSCA) platform (http://bioinfo.life.hust.edu.cn/GSCA/) was employed to comprehensively analyze the distribution of copy number variations (CNVs) of the *RAB5B* gene (18), along with the potential relationship between these CNVs and *RAB5B* mRNA expression levels. This included evaluating the specific effects of different CNVs on the *RAB5B* gene. The GSCA platform was further used to analyze differences in *RAB5B* gene methylation status across various cancer types, and to assess the correlation between methylation degree and *RAB5B* mRNA expression levels. Additionally, living conditions were compared between hypermethylated and hypomethylated patients. To quantify the correlation strength between *RAB5B* mRNA expression and CNV or methylation level, Spearman correlation analysis was conducted, with the *P* value corrected according to the false discovery rate (FDR). Furthermore, a t-test was applied to estimate differences in methylation levels between different cancers, and the *P* value was adjusted according to FDR. Finally, a log-rank test was performed to determine statistical differences in survival indicators such as OS, DSS, and PFS among groups.

### 2.5 Prediction and analysis of immunotherapy

Somatic mutation data was downloaded (initially could be rephrased to were downloaded for consistency with plural “data” in broader grammatical contexts, but “data” is often treated as singular in scientific contexts; here, keeping the original singular form to match the past tense transformation of the sentence) from the TCGA database (https://tcga.xenahubs.net/) and was analyzed using the “maftools” package in R to calculate the TMB and MSI of each tumor sample in TCGA (19). Spearman’s rank correlation coefficient was used to evaluate the correlation between *RAB5B* gene expression levels and TMB and MSI, and this relationship was visualized in the form of a radar chart using the “gradar” package in R. In immunotherapy, patients were categorized into four groups based on their treatment outcomes: disease progression (PD), disease stability (SD), complete remission (CR), and partial remission (PR). To further investigate the impact of *RAB5B*, the “Surv Miner” R package was used to determine the optimal *RAB5B* expression threshold, and two related immunotherapy cohorts were divided into low *RAB5B* expression and high *RAB5B* expression groups. Subsequently, the survival time and treatment response of these two groups were calculated. Additionally, the expression levels of MMR genes, including MLH1, MSH2, MSH6, PMS2, and EPCAM, were analyzed in different cancers, and the correlation between these MMR genes and *RAB5B* was further discussed. Finally, the “tidyverse” and “ggnewscale” packages in R were used to generate a heatmap to visually present these analysis results.

### 2.6 Evaluate the effect of *RAB5B* expression on immunity

The ESTIMATE method was applied to quantify immune and stromal cell infiltration in malignant tumor tissues. Based on the Illumina platform, the “limma” and “ESTIMATE” packages in R were utilized to conduct an in-depth analysis of correlations between *RAB5B* expression and immune scores, stromal scores, and ESTIMATE total scores across individual tumor samples. Concurrently, immune cell infiltration patterns were evaluated across diverse tumor types. To comprehensively characterize the relationship between *RAB5B* and immune cell infiltration, the TIMER 2.0 tool was employed to accurately compute immune cell infiltration scores from the TCGA database. These scoring data were systematically collected and analyzed to elucidate potential associations between *RAB5B* expression and immune infiltration dynamics. Additionally, a curated list of 150 immune-related genes was obtained from TISDB resources (20), encompassing critical categories such as MHC molecules, immunosuppressive factors, chemokine receptors, immune activators, and chemokine proteins. Subsequent analyses leveraged this gene set to calculate correlations between *RAB5B* expression and immune-related gene expression profiles using R packages (“limma”, “pheatmap”, “ggplot2”), enabling a deeper, multi-dimensional understanding of their interrelationships.

### 2.7 Biological significance analysis of *RAB5B* expression

GSEA and GSVA were applied to systematically assess the functional role of *RAB5B* in tumor biology. The analysis utilized R packages including “tidyverse”, “limma”, “org.Hs.eg.db”, “gseaplot2”, and “clusterProfiler”, incorporating C2 and C5 gene sets from the Molecular Signatures Database. Standardized enrichment scores were computed to compare false discovery rate (FDR)-adjusted differences between low- and high-*RAB5B* expression groups across diverse cancer types. Samples were stratified into high- and low-expression cohorts based on median *RAB5B* expression levels, with GSVA scores generated for all analyzed cancer types. The GSVA workflow further integrated R packages such as GSVA, GSEABase, ggthemes, BiocParallel, and visualization tools to ensure analytical rigor and comprehensive interpretation of results.

### 2.8 Correlation between *RAB5B* expression and drug sensitivity

The CellMiner database (http://discover.nci.nih.gov/cellminer/) revealed the possible close relationship between *RAB5B* expression and drug response. This database was specifically designed for cancer researchers, integrating and analyzing molecular and pharmacological data from the NCI-60 cancer cell line - a critical resource for anticancer drug screening. The processed dataset from CellMiner was made available for download, containing RNA-seq-based RNA expression data and drug activity profiles in the NCI-60 panel.For subsequent analyses, the R package “limma” was employed to filter drug datasets, retaining only FDA-approved compounds or those in clinical trials, while excluding columns with over 80% missing values. The filtered data were visualized using “ggplot2” and “ggpubr” packages, applying a significance threshold of P < 0.05.Additionally, Autodock4 software was utilized to perform molecular docking simulations, evaluating methotrexate’s binding energy and interaction patterns with *RAB5B* protein structures obtained from CellMiner. The methotrexate structure was sourced from PubChem (https://pubchem.ncbi.nlm.nih.gov/), while the *RAB5B* protein’s 3D structure was predicted using AlphaFold (https://alphafold.ebi.ac.uk/). Finally, Pymol software was employed to visualize the docking models, providing intuitive insights into methotrexate-*RAB5B* interactions.

### 2.9 Cell culture and transfection

MCF-7 cells (Cell Bank of Chinese Academy of Sciences) were cultured in DMEM (Procell, Cat# CM10033) with 10% FBS (Procell, Cat# FSP500) at 37°C/5% CO_2_. Cells were transfected at 60-70% confluency using Lipofectamine 3000 (Invitrogen, Cat# L3000015) with 50 nM *RAB5B*-targeting siRNA or negative control siRNA (GenePharma; sequences in **Supplementary Table S2**) in Opti-MEM (Gibco, Cat# 31985062). Experimental groups included: Untransfected Control, si-NC, and si-*RAB5B*. Data represent Mean ± SEM (Standard Error of the Mean), The SEM is calculated by dividing the standard deviation of the control group values in biological replicates by the square root of the sample size (√n).

### 2.10 Reverse transcription-quantitative polymerase chain reaction (RT-qPCR)

Total RNA was extracted with TRIzol (Takara, Cat# 9108), quantified via NanoDrop 2000 (Thermo Scientific), and reverse-transcribed using PrimeScript RT Master Mix (Takara, Cat# RR036A). qPCR used SYBR Premix Ex Taq II (Takara, Cat# RR820A) on a QuantStudio 3 system (Applied Biosystems) with primers (**Supplementary Table S1**). ACTB served as the reference gene. Relative expression was calculated by the 2^(−ΔΔCT) method. Statistical significance (p<0.05) was determined by one-way ANOVA with Tukey’s test (GraphPad Prism v9) across three biological replicates.

### 2.11 Cell viability determination

Cells (2×10^4^/well in 96-well plates) were treated with CCK-8 reagent (GLPBIO, Cat# GK10001) at 24h/48h post-seeding. Absorbance (450 nm) was measured using a BioTek Synergy H1 plate reader. Data were background-corrected and normalized to Untransfected Control (24h). Significance (p<0.05) was assessed by two-way ANOVA with Šídák’s test (three biological replicates, n=6 technical replicates/group).

### 2.12 Colony formation

Cells (1,000/well in 6-well plates) were cultured for 7 days (medium refreshed every 3 days), fixed with methanol, and stained with 0.5% crystal violet (Beyotime, Cat# C0121). Colonies (>50 cells) were scanned (Epson Perfection V600) and counted using ImageJ (v1.53t). Statistical analysis used unpaired t-tests (three biological replicates).

### 2.13 Cell scratch healing analysis

Confluent cells in 6-well plates were scratched with a sterile pipette tip, washed with PBS, and cultured in 2% FBS/DMEM. Wound images (T0/T48h) were captured at identical locations using a Nikon Ti-E microscope (DS-Fi3 camera, NIS-Elements v5.30). Wound area was measured in ImageJ (v1.53t), with closure percentage calculated as [(Area_T0 − Area_T48)/Area_T0] × 100%. Statistical significance (p<0.05) was determined by unpaired t-tests (three biological replicates, n=3 scratches/group).

### 2.14 Statistical analysis

In order to carry out the verification of bioinformatics, firstly, the obtained dataset was strictly screened, and the missing values and repetitive results were eliminated to ensure the accuracy and reliability of the data. Subsequently, the TPM value in the filtered dataset was transformed by log2 (TPM+1) for subsequent analysis. The Mann-Whitney U test (also called the Wilcoxon rank sum test) was used to compare the expression level of *RAB5B* between normal tissues and tumor tissues to verify whether the difference between them was statistically significant. For the data from the CCLE database covering different organizational sources, the Kruskal-Wallis method was used to comprehensively analyze the expression of *RAB5B*. When comparing the heterogeneous expression level of *RAB5B* between different groups or between tumors and normal tissues, the choice of analysis tool—paired t-test or unpaired t-test—depended flexibly on whether the samples were paired. In the whole analysis process, the significance level was set to a p-value less than 0.05 to ensure the accuracy and reliability of the results. All analysis work was done in R software (version 4.4.0, https://www.R-project.org).

## 3 Result

### 3.1 Analysis of the differential expression of *RAB5B* gene in cancer

**Figure 1:**
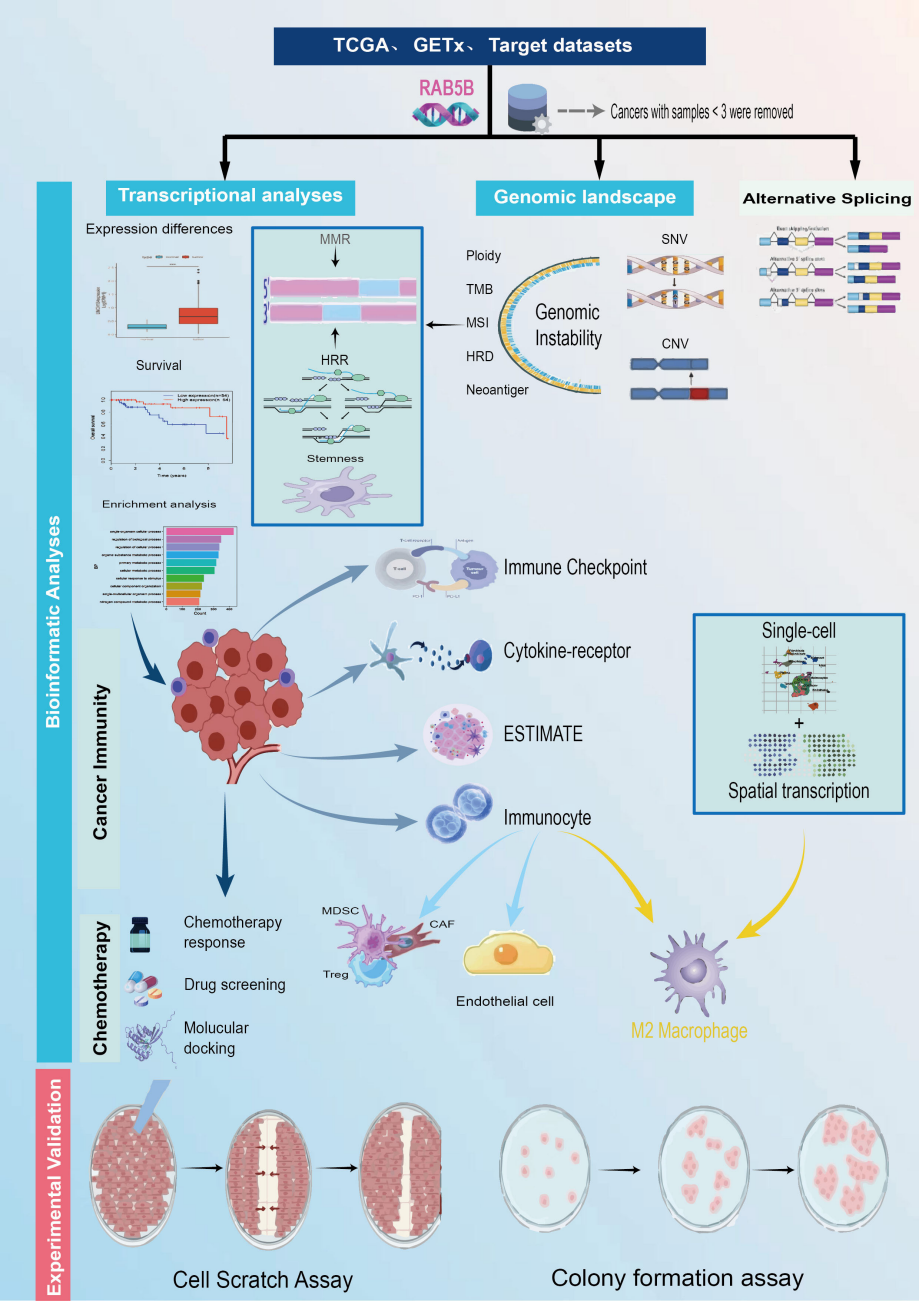
The study’s methodological approach outlines an extensive examination of the target gene *RAB5B* across numerous cancer types. First, the expression levels of *RAB5B* were assessed on a pan-cancer scale, followed by an in-depth analysis of its relationships with different cancer subtypes. The study compared variations in *RAB5B* expression across 33 types of malignant and non-malignant tissues, as well as across various cellular contexts. Subsequent steps involved single-cell transcriptomic analyses and prognostic assessments. A detailed analysis of the genomic landscape was conducted, focusing on genomic instability, utilizing data from the cBioPortal and GSCA databases to evaluate pan-cancer alterations, such as CNVs and DNA methylation patterns. Additionally, the study explored the correlation between *RAB5B* expression and factors like TMB, MSI, and MMR status. To elucidate the functional role of *RAB5B* in cancer development, the research delved into its involvement in immune modulation, assessing sequence function enrichment, regulation of immune checkpoints, interactions with cytokine receptors, and immune cell infiltration. Finally, the potential therapeutic relevance of *RAB5B* was evaluated through predictions of chemotherapy responses, drug sensitivity analyses, and relevant experimental assessments.

**Figure 2:**
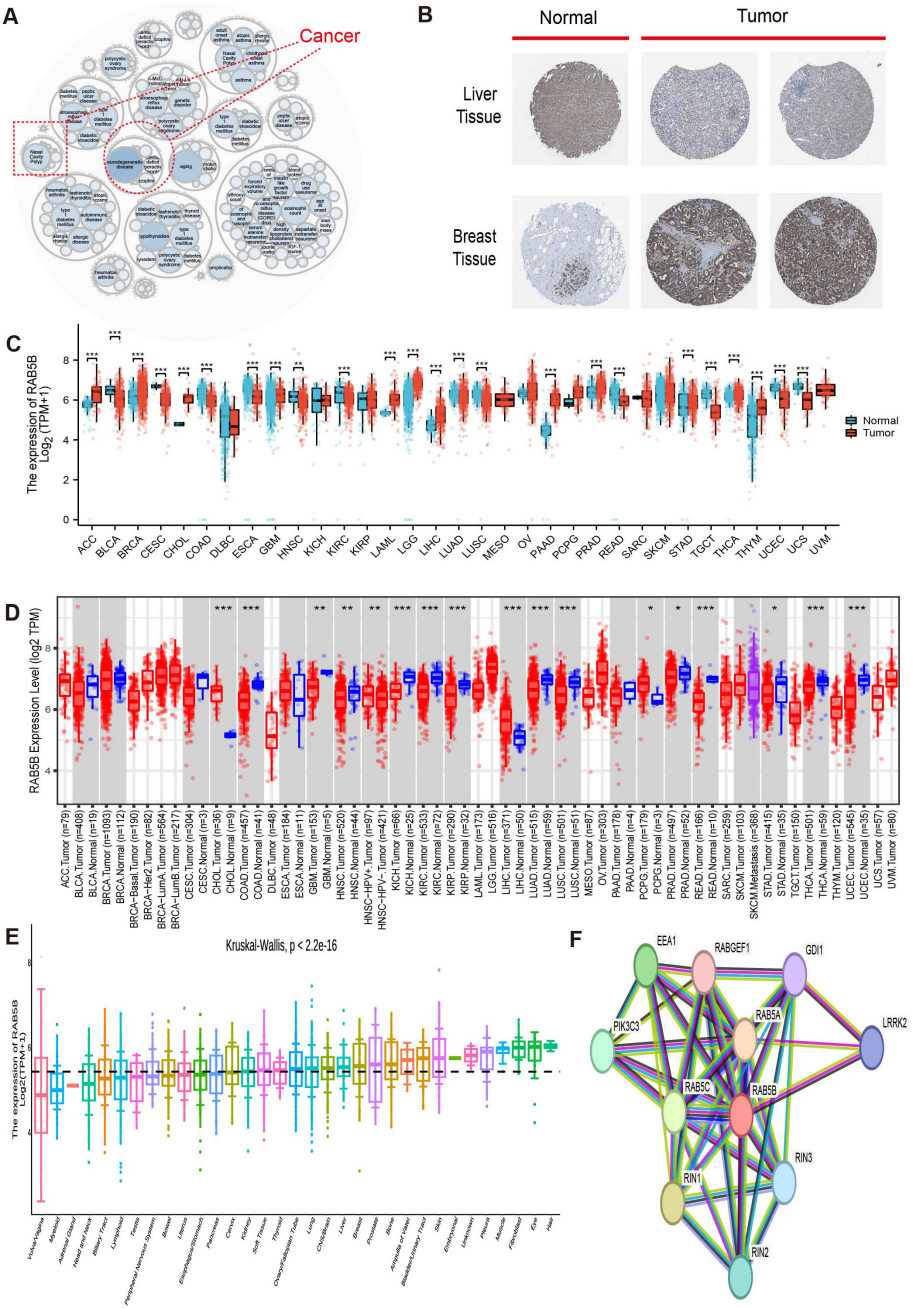
*RAB5B* expression in human malignancies. **(A)** Utilizing the openTarget online platform, diseases associated with *RAB5B* were reviewed, with a dotted red box highlighting cancers related to *RAB5B*. **(B)** Protein expression of *RAB5B* in normal (left) and tumor (right) tissues was compared through immunohistochemical staining, involving lymph node and breast tissues. **(C)** *RAB5B* expression differences between tumor (TCGA data: 33 cancer types, n=10,534) and normal tissues (GTEx data: 31 tissue types, n=7,568) were evaluated using standardized bioinformatics pipelines to ensure reproducibility and transparency in data integration and analysis. (Statistical significance markers were *p-values<0.05, **p-values<0.01, ***p-values<0.001). **(D)** The expression status of *RAB5B* mRNA in various cancers was examined using the TIMER2.0 platform, with tumor tissues marked in red and normal tissues in blue.(Statistical significance markers were *p-values<0.05, **p-values<0.01, ***p-values<0.001). **(E)** The expression status of *RAB5B* in 33 types of tissues was analyzed using CCLE data, with different colors employed to distinguish various cancer types in the visualization. **(F)** A PPI network revealed proteins that may interact with *RAB5B*, with different colored lines representing different prediction methods.

**Figure 1** illustrates the overall scope of the study. RAB5B-related diseases are explored on openttarget, and the bubble diagram shows that RAB5B is related to nasal polyps and neurodegenerative diseases (**Figure 2A**). As shown in **Figure 2**, it can be seen intuitively that the expression of *RAB5B* protein in LIHC and BRCA is significantly higher in cancer tissues than in normal tissues. In order to explore the significance of *RAB5B* in malignant tumors, the expression level of *RAB5B* was carefully examined in 33 cancer types using comprehensive data sets obtained from TCGA and GTEx databases. The results of the study demonstrated that the expression level of the *RAB5B* gene was significantly up-regulated in ACC, BRCA, CHOL, LAML, LGG, LIHC, PAAD, PRAD, THCA and THYM. Conversely, the expression of *RAB5B* was significantly down-regulated (decreased) in BLCA, CESC, COAD, ESCA, READ, TGCT, UCEC and UCS when compared to their respective normal tissue counterparts (**Figure 2C**), that may be attributed to specific tumor microenvironment factors, genetic mutations, or the presence of different regulatory mechanisms affecting *RAB5B* expression in these particular cancers. Subsequently, the difference of *RAB5B*mRNA between cancer tissues and normal tissues was analyzed by TIMER2.0 with tumor tissues marked in red and normal tissues in blue *RAB5B* mRNA was significantly increased in three kinds of cancers: CHOL, LIHC, and PCPG and significantly decreased in COAD, KICH, KIRC and READ as shown in **Figure 2D**. In addition, the public documents of *RAB5B* expression were downloaded from CCLE database, and the differences of *RAB5B* expression in 33 organizations were studied. **Figure 2E** illustrates the widespread expression of *RAB5B* across diverse tissues, with its expression upregulated in most tumor types except vaginal cancer and myeloma. Different cancer types were distinguished using color coding for clarity. Final analysis involved constructing a PPI network using data from the GeneMANIA database (21), which identified the top 10 proteins demonstrating the strongest correlation with *RAB5B*. **Figure 2F** shows a protein interaction network diagram. From the diagram and correlation analysis, it can be seen that there is a strong physical interaction between *RAB5B* and protein such as RAB5A, RAB5C and RIN3. Simultaneously, this analysis elucidated potential signal pathways involving *RAB5B* and its molecular interactions, offering insights into the mechanisms through which *RAB5B* influences gene expression and cellular behavior.

### 3.2 Analysis of single cell expression of *RAB5B* in different cells of TME

**Figure 3:**
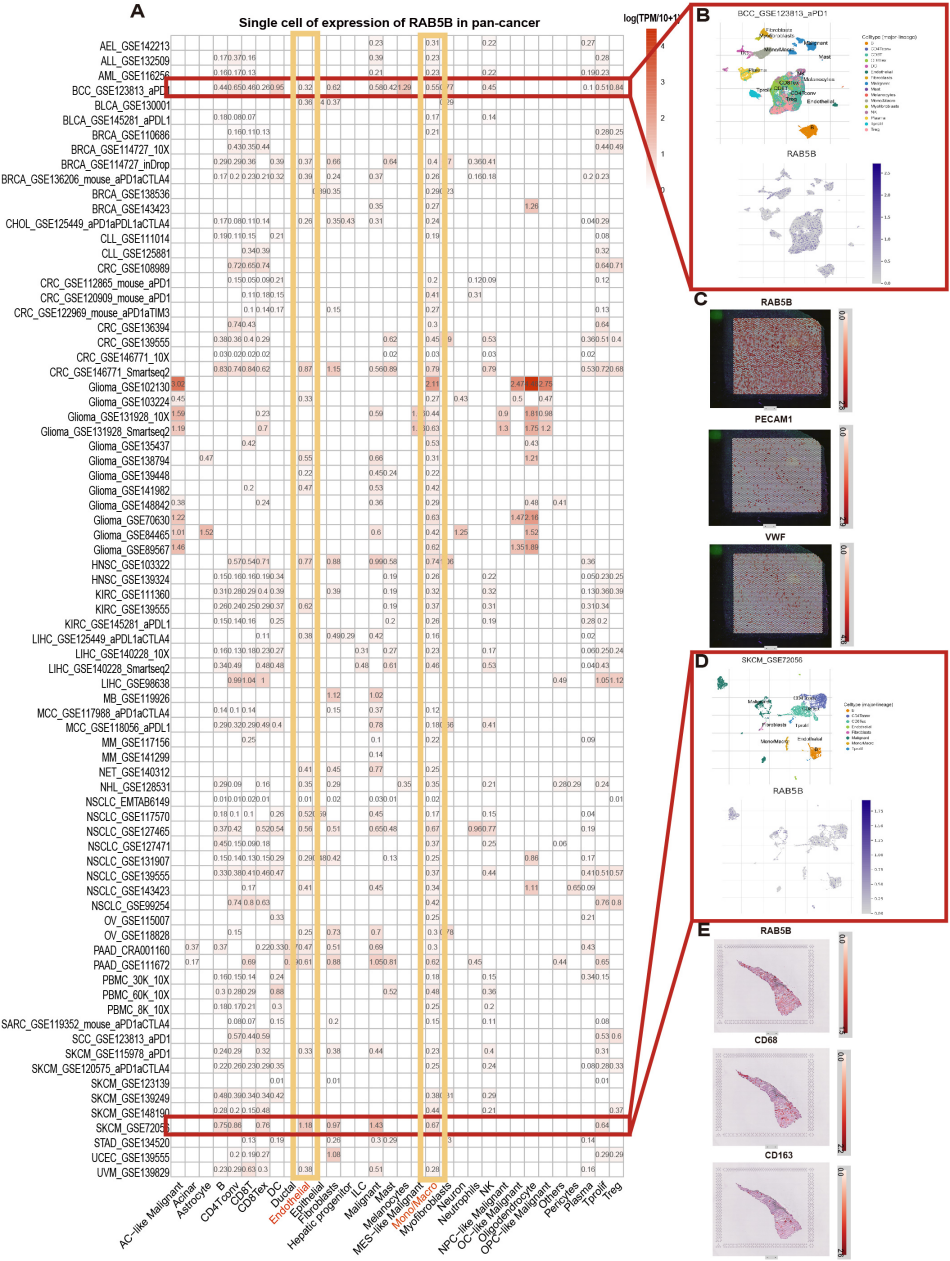
Cell type distribution and spatial expression characteristics of *RAB5B* expression. **(A)** The *RAB5B* expression across 33 cell types was summarized using aggregated single-cell data from 77 studies, which specified patient/tissue cohorts for clinical/bioinformatic figures. **(B)** A scatter plot displayed the distribution of six different cell types and their *RAB5B* expression levels in the GSE123813 BCC dataset. **(C)** Spatial transcriptome sections showed the spatial expression of *RAB5B*, PECAM1, and VWF markers, with dot colors indicating marker expression levels. **(D)** A scatter plot displayed the distribution of 13 different cell types and their *RAB5B* expression levels in the GSE72056 SKCM dataset. **(E)** Spatial transcriptome sections showed the spatial expression of *RAB5B*, CD68, and CD163 markers, with dot colors indicating marker expression levels.

To gain insights into the primary cell types expressing *RAB5B* in CME, an analysis of *RAB5B* expression was conducted at the single-cell level across a dataset comprising 77 cancer samples. The thermogram depicted in **Figure 3A** shows that the expression levels of RAB5A in 32 cell types were analyzed in 79 data sets by using the TISCH network tool. The results showed that *RAB5B* was mainly expressed in endothelial cells and immune cells (especially monocytes/macrophages). Notably, in GSE 123813 data set containing 86 BCC patients, *RAB5B* is mainly expressed in B cells, CD4Tcon, Treg and other cells (**Figure 3B**). At the same time, in GSE 72056 skin melanoma data set containing 4645 single cell samples, *RAB5B* was highly expressed in depleted Endothelial cell and monocytes in skin melanoma microenvironment (**Figure 3D**). In addition, the spatial transcription data on SpatialDB showed that *RAB5B* spatially overlapped with endothelial cell markers PECAM1 and VWF in BCC cancer tissues and M2 macrophage markers CD68 and CD163 in skin melanoma tissues (**Figures 3C, E**). The similar spatial distribution of these markers indicated that *RAB5B* might be co-expressed with CD68, CD163, PECAM1 and VWF in these cell types.

### 3.3 Analysis of the prognostic value of *RAB5B* in different cancers

**Figure 4:**
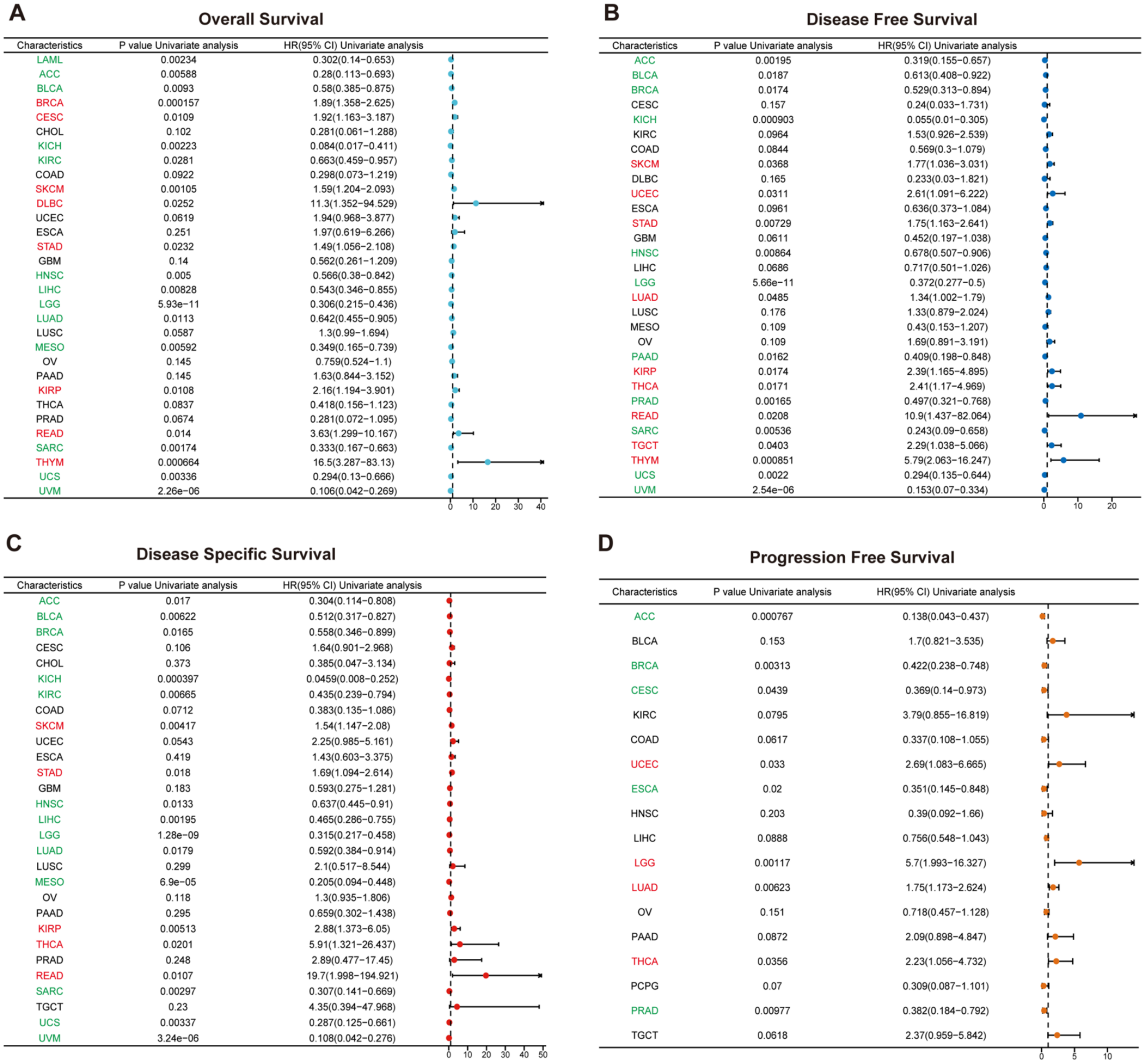
Prognostic value of *RAB5B* expression in pan-cancer via one-dimensional cox regression analysis. Forest plots using the Cox proportional-hazards model with Bonferroni correction illustrated the associations between *RAB5B* expression and OS **(A)**, DFS **(B)**, DSS**(C)**, and PFS **(D)**. HR values >1 indicated risk factors (red), while HR values <1 indicated favorable factors (green).

For each cancer type, one-way Cox regression analysis was utilized, based on data from the TCGA database, to investigate the association between *RAB5B* expression levels and the prognosis of various cancers. Survival analyses were conducted for OS, DSS, DFS and PFS. As illustrated in **Figure 4**, a significant correlation between *RAB5B* and the prognosis of most cancers is evident. COX proportional hazard model was used to analyze the prognosis of all cancers. The forest map of **Figure 4A** shows that in BRCA, CESC, SKCM, DLBC, STAD, KIRP, READ and THYM, the expression of *RAB5B* is positively correlated with OS, but negatively correlated with LAML, ACC, BLCA, KICH, KIRC, HNSC, LIHC, LGG, LUAD, MESO, SARC, UCS and UVM. In DFS, the expression of *RAB5B* was positively correlated with SKCM, UCEC, STAD, LUAD, KIRP, THCA, READ, TGCT and THYM. In addition, *RAB5B* can protect DFS of ACC, BLCA, BRCA, KICH, HNSC, LGG, PAAD, PRAD, SARC, UCS and UVM (**Figure 4B**). To exclude non-cancer-related deaths and better reflect the impact of cancer treatment, an additional DSS analysis was conducted. It was found that *RAB5B* was regarded as a protective factor in ACC, BLCA, BRCA, KICH,KIRC,HNSC, LIHC, LGG, LUAD, MESO, SARC, UCS and UVM, but as a risk factor in SKCM, STAD, KIRC, THCA and READ (**Figure 4C**). Notably, *RAB5B* emerges as a risk factor for both LGG and LUAD in the PFS forest plot analysis, while concurrently functioning as a risk contributor to UCEC and THCA. Conversely, it exhibits protective properties in ACC, BRCA, CESC, and ESCAPRAD. The CNV distribution of the *RAB5B* gene across distinct cancer types is illustrated using pie charts, with color-coding distinguishing different CNV categories. Notably, *RAB5B* emerges as a risk factor for PFS in LGG and LUAD through forest plot analyses. Additionally, elevated *RAB5B* expression correlates with poor prognosis in UCEC and THCA. Conversely, *RAB5B* functions as a protective factor in ACC, BRCA, CESC, and ESCA, highlighting its context-dependent role in oncogenic outcomes. (**Figure 4D**).

### 3.4 Characteristics of *RAB5B* gene alterations in pan-cancer and their association with genomic instability: a multi-omics analysis

**Figure 5:**
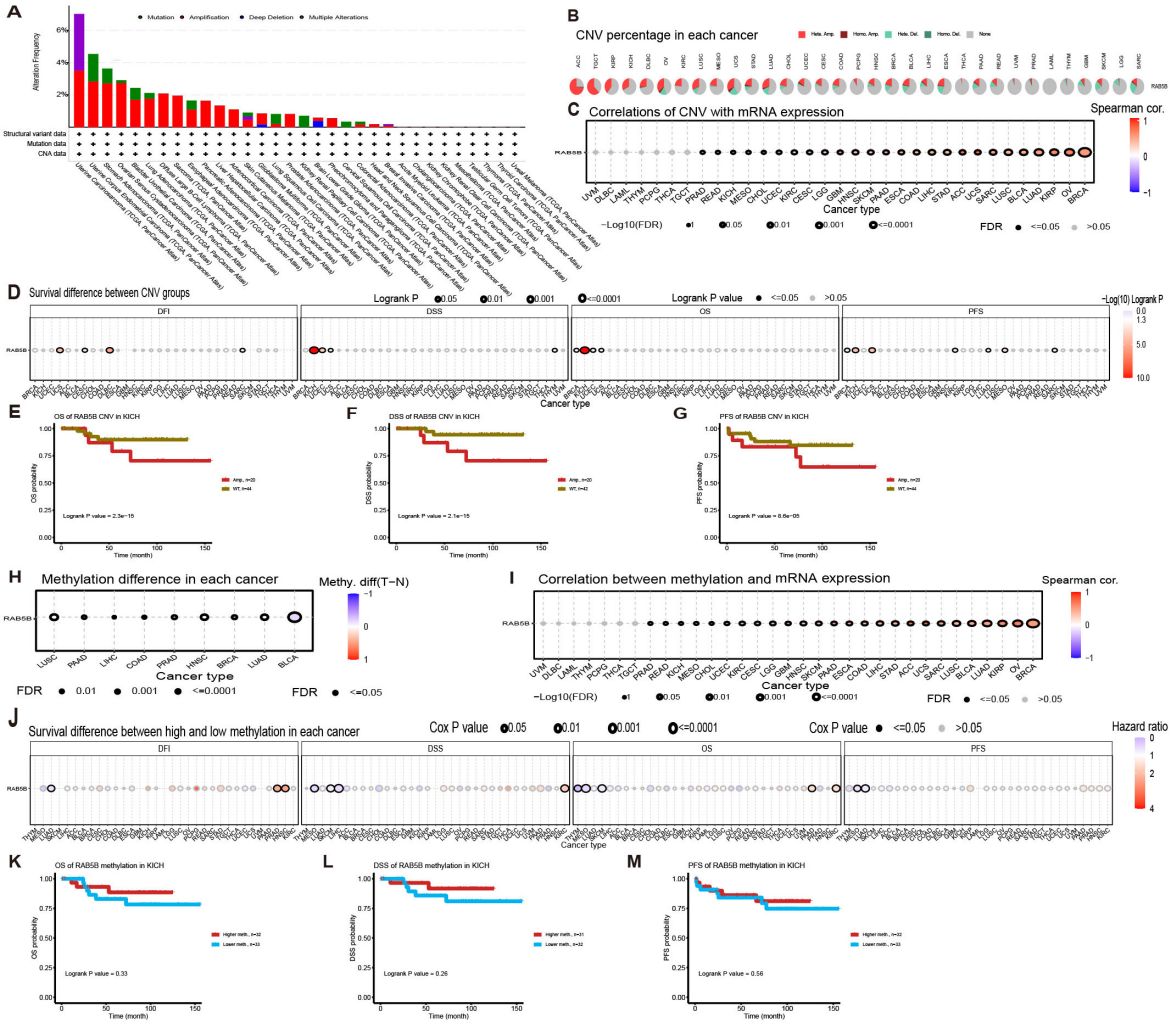
Association of *RAB5B* with genomic instability in TCGA tumors. **(A)** Genomic changes in *RAB5B*, including mutations, amplifications, deep deletions, and multiple modifications, were examined in the TCGA pan-cancer cohort. **(B)** A CNV summary displayed the CNVs of the *RAB5B* gene across various cancer types. **(C)** The relationship between *RAB5B* CNV and mRNA expression was explored through Spearman correlation analysis. **(D)** The correlations between *RAB5B* CNV status and OS, DSS, PFS, and DFI were analyzed. **(E–G)** Kaplan-Meier survival curves generated using the GSCA web tool assessed the prognostic value of *RAB5B* CNV in ACC patients. **(H)** A methylation difference analysis was performed for cancers with more than 10 tumor-normal sample pairs, displaying only results with *p*-values ≤0.05. **(I)** A Spearman correlation analysis investigated the relationship between *RAB5B* methylation and mRNA expression. **(J)** The correlations between *RAB5B* methylation status and OS, DSS, PFS, and DFI were analyzed. **(K–M)** Kaplan-Meier survival curves depicted survival outcomes in ACC patients stratified by high/low RAB5B methylation status, with sample sizes annotated in the figure legend and specific statistical tests/significance thresholds noted.

The occurrence of many cancers often stems from the variation of genome (22). Through in-depth exploration of the cBioPortal database, genomic alterations of the *RAB5B* gene across various cancer types were identified. Specifically, as shown in **Figure 5A**, “amplification” is the main type of *RAB5B* variation in patients with UCS, and the frequency of this variation is the highest, exceeding nearly 4%. Additionally, analysis revealed variations in *RAB5B* gene mutation frequency across different cancer types. **Figure 5B** also presents the analysis results of CNV and methylation status of *RAB5B* gene in GSCA database. The CNV profile of the *RAB5B* gene across different cancer types is visualized through pie charts, where distinct color coding differentiates various CNV categories.The legend part details the CNV types represented by each color, including the percentage of heterozygous amplification, heterozygous deletion and CNV-free samples, among which heterozygous amplification accounts for the most. **Figure 5C** shows the correlation between CNV and mRNA expression. In CHOL, GBM, PAAD, ESCA, ACC, UCS, KIRP, OV and BRCA, there is a substantial positive correlation between the expression of *RAB5B* CNV and mRNA. On the contrary, this relationship is not significant in UVM, DLBC, LAML, THYM, PCPG, THCA, TGCT and PRAD. The differential impact of *RAB5B* gene CNV and generalized expression patterns on survival outcomes across cancer types was analyzed. Samples were stratified into subgroups based on CNV levels determined by GSCA, as illustrated in **Figure 5D**. Results indicated that elevated *RAB5B* CNV levels correlated with reduced overall survival in specific cancers, including UCS, KICH, and UCEC. Notably, KICH patients in the high-CNV subgroup exhibited consistently poor prognostic indicators across all evaluated survival metrics (**Figures 5E–G**). DNA methylation is a chemical modification process, which may lead to the inactivation of oncogenes, and then may promote the occurrence of cancer. As shown in **Figure 5H**, in-depth analysis of methylation differences between normal and tumor samples reveals that methylation levels in tumor samples of various cancer types, including LUSC, PAAD, LIHC, COAD, PRAD, HNSC, BRCA, LUAD, and BLCA, are generally elevated compared to corresponding normal samples. Notably, BLCA samples exhibit the highest methylation levels among these cancer types. Regarding the relationship between DNA methylation and *RAB5B* expression, a positive correlation is observed across all cancer types except UVM, especially in CHOL, GBM, PAAD, ACC, UCS, KIRP,OV etc (**Figure 5I**). Based on the median methylation level of tumor samples, they are categorized into hypermethylation and hypomethylation groups. Analysis further indicates that hypermethylation of the *RAB5B* gene correlates with overall survival rates in PRAD, HNSC, and KIRC patients, as depicted in **Figure 5J**. Notably, across various survival indicators, a consistent trend emerges: in KICH patients, high methylation levels of the *RAB5B* gene are associated with a lower risk of death, clearly illustrated in **Figures 5K–M**.

### 3.5 Co-expression analysis of *RAB5B* and immune-related genes

**Figure 6:**
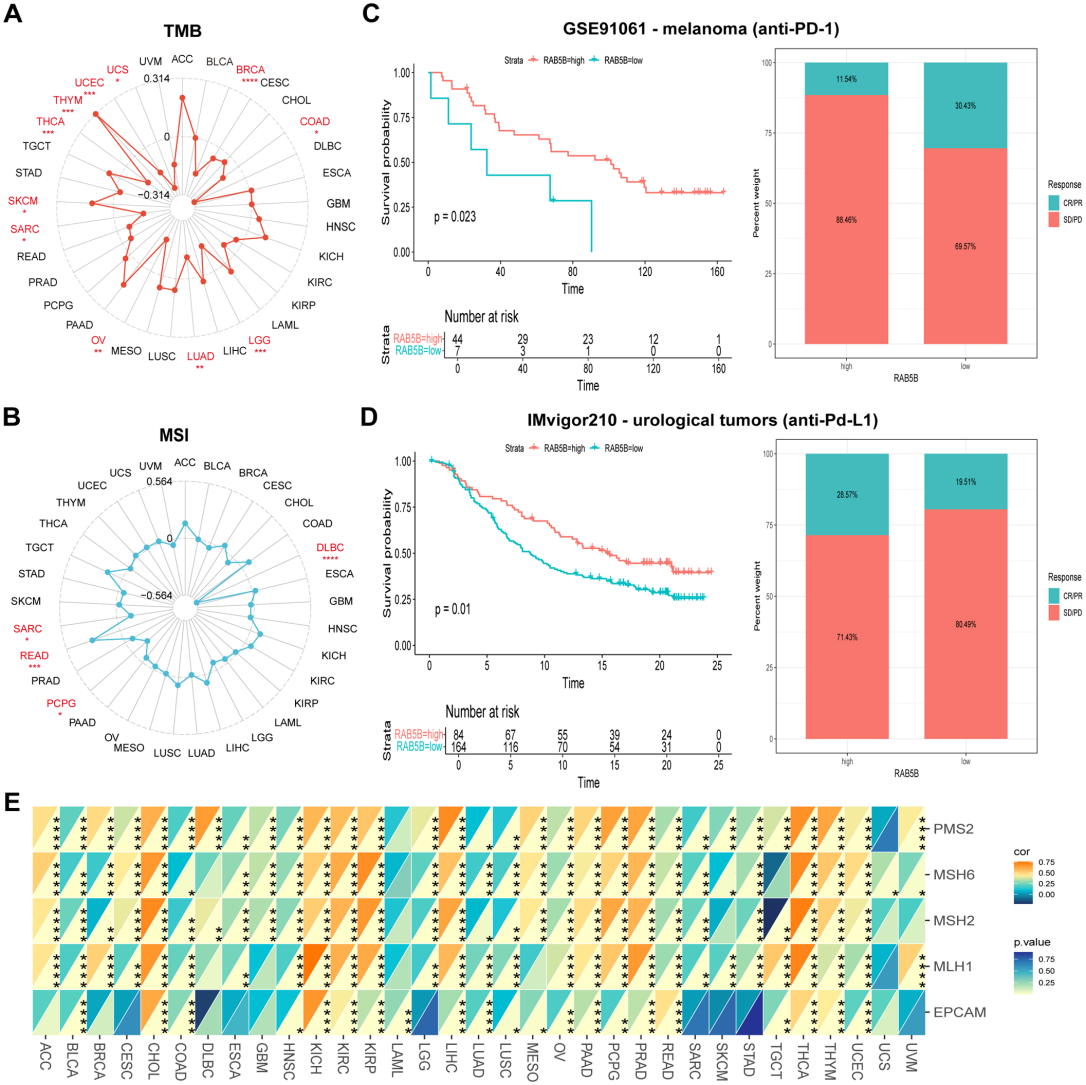
Association of *RAB5B* expression with TMB, MSI, and MMR in different cancer types. radar charts were used to visualize the relationships between *RAB5B* expression and TMB **(A)** and MSI **(B)**. **(C, D)** Kaplan-Meier curves and stacked bar charts displayed the survival outcomes and immunotherapy response proportions of low and high PDIA3 patient groups in two independent cohorts GSE91061 (top) and IMvigor210 (bottom). **(E)** A heatmap displayed the correlations between *RAB5B* expression and five MMR genes in a pan-cancer cohort. Statistical significance was marked as *p < 0.05, **p < 0.01, ***p < 0.001 and ****p < 0.0001.

TMB and MSI are the key indexes to evaluate the efficacy of immune checkpoint inhibitors, which have a significant impact on the prognosis and treatment response of patients (23, 24). Specifically, TMB is determined by counting the number of somatic non-synonymous mutations in the coding region of tumor genome, while MSI is evaluated by measuring the mutation frequency caused by the defect of MMR mechanism per million base pairs. MMR plays a central role in correcting errors in DNA replication during cell division. When MMR gene expression is down-regulated or its function is impaired, mutations may occur in somatic cells. As shown in **Figures 6A, B**, the expression level of *RAB5B* gene is positively correlated with TMB in SKCM and THYM. However, in BRCA, COAD, LGG, LUAD, OV, SARC, THCA, UCE and UCS, *RAB5B* was negatively correlated with TMB. Similarly, in READ, *RAB5B* is positively correlated with MSI, but in DLBC, PCPG and SARC, *RAB5B* is negatively correlated with MSI. When assessing the potential of *RAB5B* expression as a biomarker for predicting the response to immunotherapy, two patient groups were selected, one having received anti-PD-1 treatment and the other anti-PD-L1 treatment. The results showed that the high expression of *RAB5B* was associated with high survival rate (88.46%) and low response rate to anti-PD-1 therapy (only 11.54%) in patients with urinary system tumors, as shown in **Figure 6C**. In the analysis of the IMvigor210 cohort, it was observed that patients with high *RAB5B* expression exhibited greater sensitivity to immunotherapy, as illustrated in **Figure 6D**. Furthermore, an in-depth exploration of the relationship between the expression of MMR genes (comprising MLH1, MSH2, MSH6, PMS2, and EPCAM) and *RAB5B* expression was conducted, with the specific findings presented in **Figure 6E**. The results showed that there was a significant correlation between the expression level of MMR gene and the expression level of *RAB5B* in all types of tumor samples. Especially in ACC, CHOL, KICH, KIRC, KIRP, LIHC, PCPG, PRAD, THCA and TYHM, the expression of MMR gene was positively correlated with the expression of *RAB5B*. These findings further strengthen the possibility of *RAB5B* as a potential biomarker to evaluate the stability of CHOL genome.

### 3.6 Study on the correlation between *RAB5B* and immune related factors

**Figure 7:**
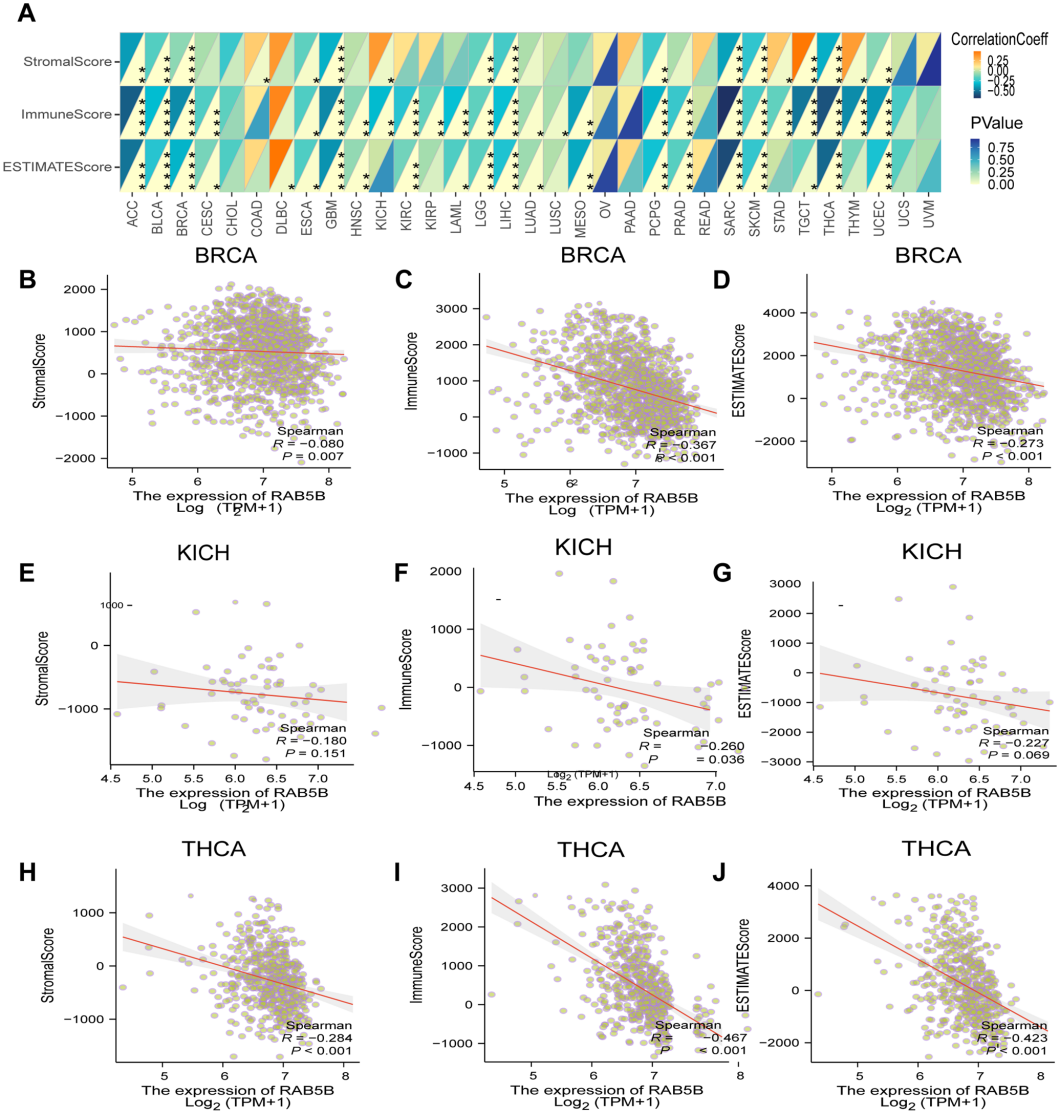
Analysis of *RAB5B* expression and immune characteristics. **(A)** The relationships between *RAB5B* expression and immune score, stromal score, and estimate score were studied through Spearman correlation analysis, with correlation coefficients displayed in the upper left triangle and p-values in the lower right triangle (*p < 0.05, **p < 0.01, ***p < 0.001 and ****p < 0.0001). The relationships between *RAB5B* expression and these scores in BRCA **(B–D)**, KICH **(E–G)**, and THCA **(H–J)** were presented.

TME is a complex system, including stromal cells, fibroblasts, endothelial cells and various components of innate and adaptive immune system (25). A deep understanding of the complexity of TME is very important to improve the therapeutic effect. To investigate the relationship between *RAB5B* expression and TME, a comprehensive evaluation of the matrix index, immune index, and ESTIMATE score was conducted across several tumor types.The results showed that there was a significant negative statistical correlation between the expression level of *RAB5B* and matrix index, immune index and ESTIMATE score, especially in these tumors with high scores, and only a few cancers showed positive correlation, such as COAD and DLBC (**Figure 7A**). For the specific analysis results of BRCA, KICH and THCA, please see **Figures 7B–J**.

### 3.7 Interplay between *RAB5B* expression and immune infiltration/immunoregulation

**Figure 8:**
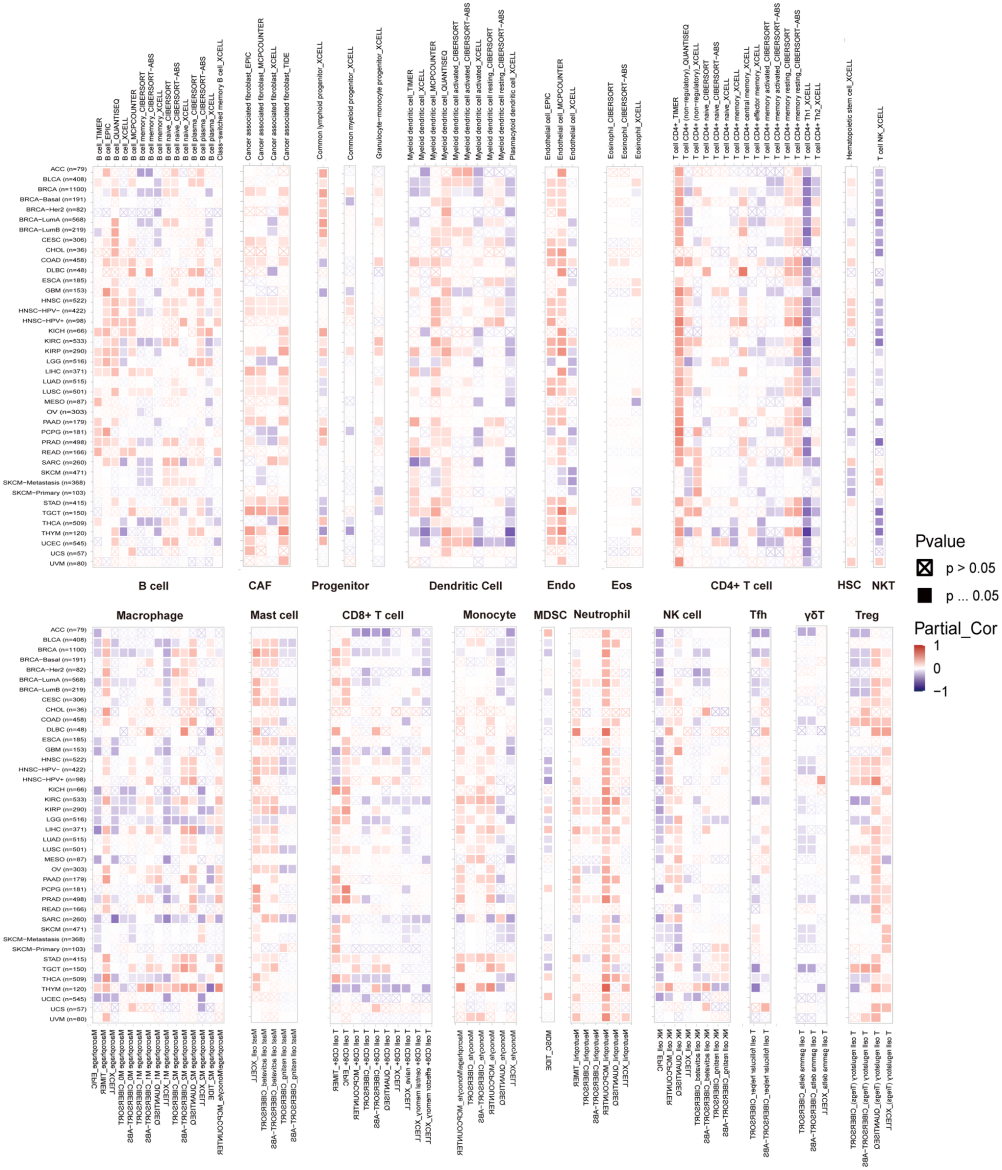
Relationship between *RAB5B* expression and immune cell infiltration levels in various malignancies. Data from the TIMER 2.0 database and TCGA illustrated the relationships between *RAB5B* expression and the infiltration levels of B cells, Cancer-Associated Fibroblasts, Progenitor cells, Dendritic cells, Endothelial cells, Eosinophils, CD4+ T cells, Hematopoietic stem cells, NKT cells, Macrophages, Mast cells, CD8+ T cells, Monocyte, Myeloid-Derived Suppressor Cells, Neutrophils, Natural killer cells, T Follicular Helper, γ/δ and T cell. Red indicated positive correlations, while blue indicated negative correlations.

**Figure 9:**
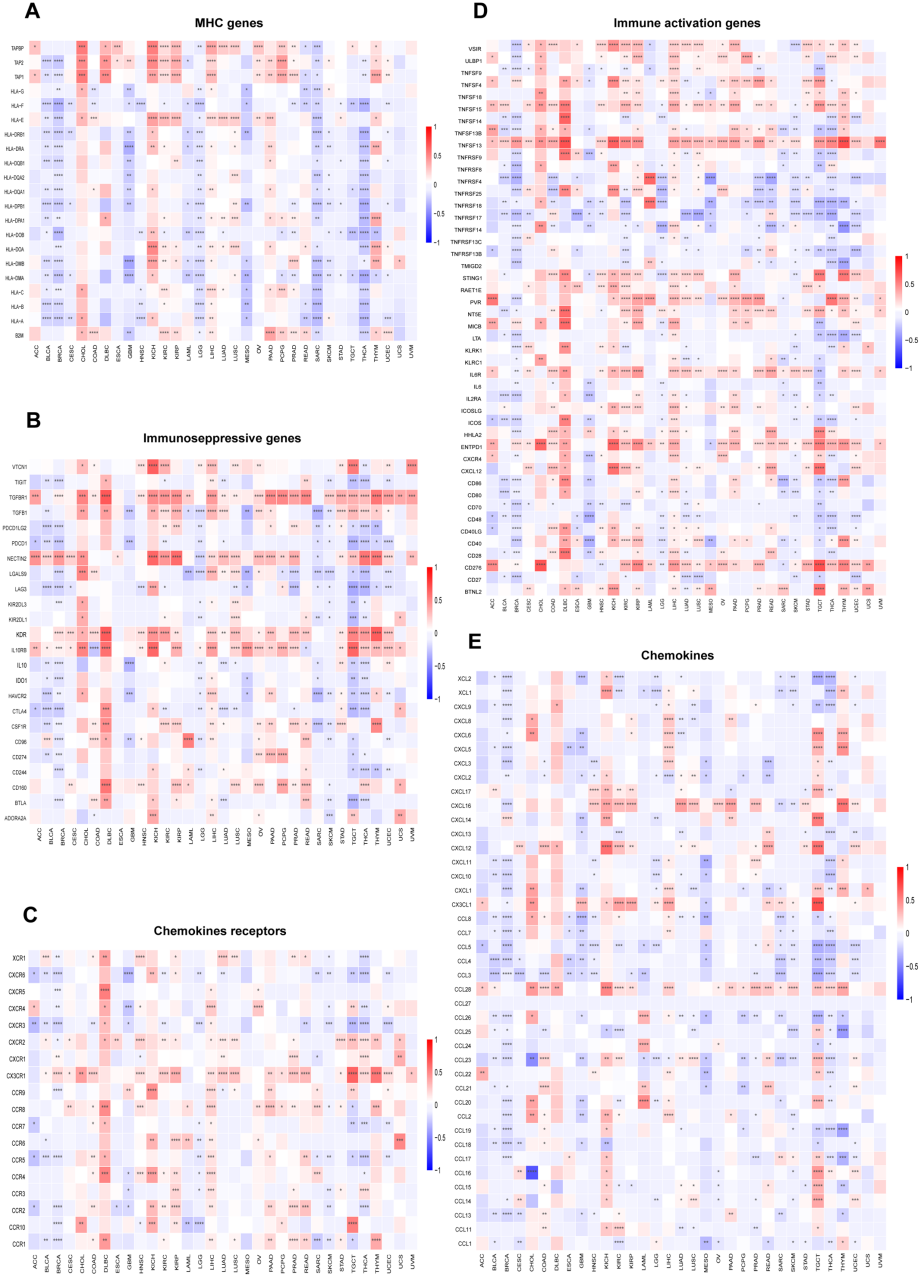
Relationship between *RAB5B* expression and immune-related genes. The relationships between *RAB5B* expression and **(A)** MHC genes, **(B)** Immunoseppressive genes, **(C)** Chemokines receptors **(D)** Immune activation genes, and **(E)** Chemokines were displayed. Statistical significance was marked as *p < 0.05, **p < 0.01, ***p < 0.001 and ****p < 0.0001.

The relationship between immune cell infiltration and *RAB5B* expression was thoroughly investigated to elucidate the association between *RAB5B* and tumor immunity. Analysis confirmed that *RAB5B* expression correlates with infiltration levels of diverse immune cell types, including: B cells, Cancer-Associated Fibroblasts, Progenitor cells, Dendritic cells, Endothelial cells, Eosinophils, CD4+ T cells, Hematopoietic stem cells, NKT cells, Macrophages, Mast cells, CD8+ T cells, Monocyte, Myeloid-Derived Suppressor Cells, Neutrophils, Natural killer cells, T Follicular Helper, γ/δ and T cell. In many tumor types, the expression of *RAB5B* is positively correlated with the infiltration level of neutrophils in B cells, Cancer-Associated Fibroblasts, Progenitor cells,endothelial cells, Dendritic Cell, CD4+ T cells,mast cells,Monocyte, Neutrophils and Treg. Recent studies have underscored the critical role of immune cells, including CD4+ T cells, CAFs, MDSCs, neutrophils, and macrophages, in shaping responses to tumor immunotherapy (**Figure 8**). The functional contributions of these immune subsets have emerged as pivotal determinants in therapeutic outcomes, necessitating their careful consideration in oncology treatment strategies. Evidence suggests that *RAB5B* modulates tumor progression, prognostic potential, and therapeutic efficacy through dynamic interactions with immune cell populations, highlighting its role as a regulatory node in immune-tumor crosstalk. This interplay underscores the significance of dissecting immune-related mechanisms when evaluating targeted interventions for cancer management.In addition, the relationship between *RAB5B* expression and immune response genes in 33 cases of tumor was discussed by co-regulation analysis of gene expression. Most of these genes encode MHC proteins, immune activation and inhibition proteins, chemokines and their receptors. The generated thermogram showed that most immune response genes were co-expressed with *RAB5B* (**Figure 9**), and were positively correlated with *RAB5B* in all tumor types except BLCA, BRCA, GBM, LGG, MESO, SARC and THCA.

### 3.8 Biological significance of *RAB5B* expression in tumors

**Figure 10:**
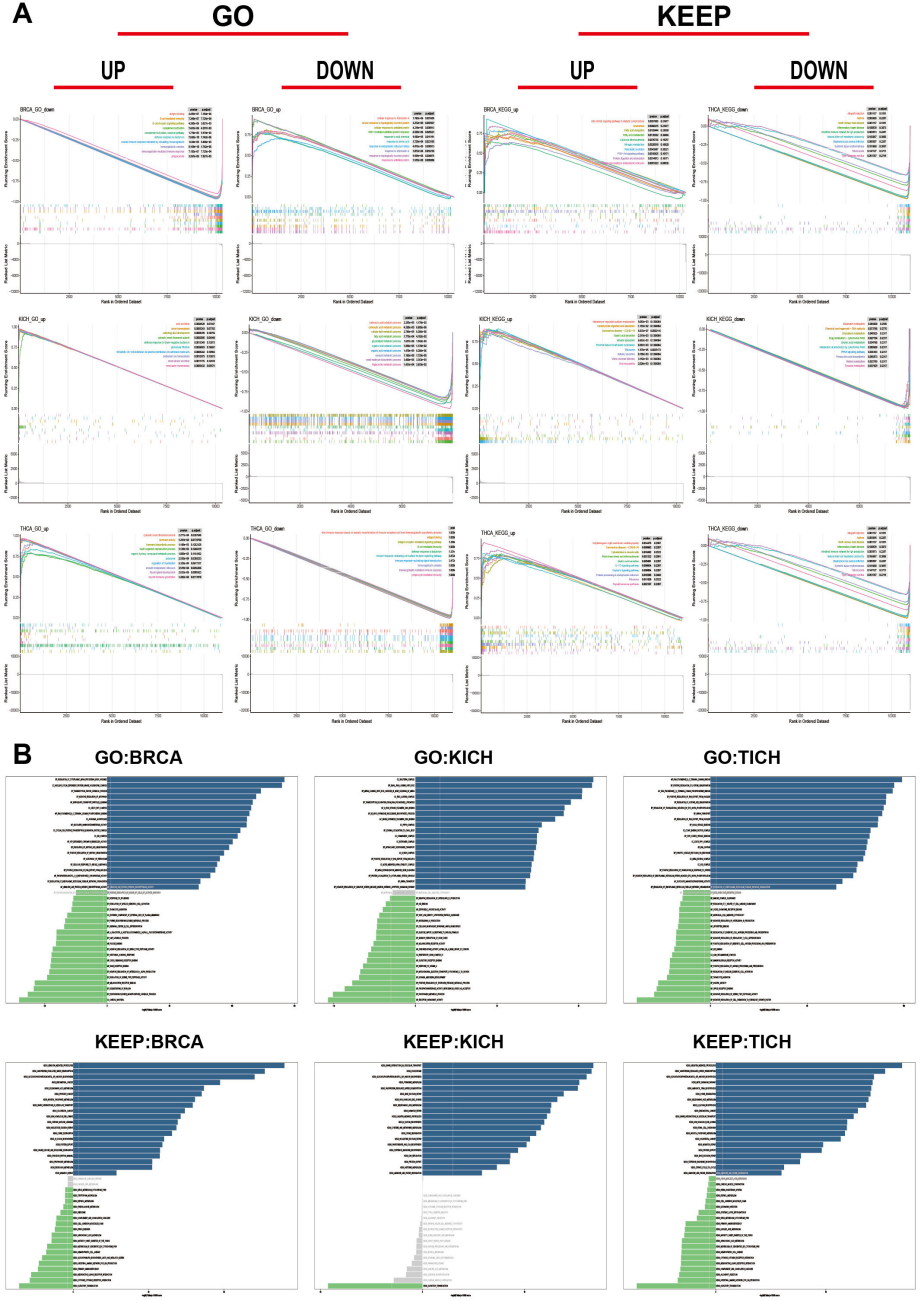
Functional analysis of *RAB5B.* **(A)** GSEA results for *RAB5B* in BRCA, ACC, and UCEC were displayed through GO functional annotation and KEGG pathway analysis. Different colored curves represented regulated functions or pathways in different types of cancers, with peaks on the curves indicating positive regulation and descending segments indicating negative regulation. **(B)** GSVA analysis results using GO and KEGG datasets were presented in BRCA, ACC, and UCEC. Blue bars indicated pathways with the most significant positive correlations, green bars indicated pathways with the most significant negative correlations, and gray bars indicated no significant correlations (FDR > 0.05). The horizontal axis represented the -log10(P-value) of GSVA scores.

Through sequence function enrichment analysis, the function of *RAB5B* in cancer was discussed. Based on the median expression level of *RAB5B* in various cancer types, samples were categorized into low and high *RAB5B* groups. Subsequent GSEA and GSVA analyses were performed to uncover the biological processes associated with *RAB5B*. It was demonstrated that *RAB5B* exerts a positive regulatory influence on various immune-related activities in BRCA, KICH, and THCA, including B cell receptor signaling pathway, humoral immune response mediated by circulating immunoglobulin, immune response mediated by mmunoglobulin, cell response to protein with incorrect topology, cell response to unfolded protein, unfolded protein response mediated by ire1, autoimmune thyroid disease, graft-versus-host response, unfolded protein response of endoplasmic reticulum and so on. In addition, many KEGG pathways, such as nitrogen metabolism, allograft rejection, thyroid hormone synthesis, etc., all show significant correlation with *RAB5B*. These findings reveal that there is a potential close relationship between the expression level of *RAB5B* in tumor microenvironment and the immune activation state. In BRCA, *RAB5B* is predicted to be a positive regulator that promotes interleukin-4 (IL-4) cellular response and cellular response to misfolded proteins. In KICH, *RAB5B* is a positive regulator of renal water homeostasis and viral myocarditis. In THCA, *RAB5B* can positively regulate thyroid gland development and thyroid hormone synthesis. However, it also plays a negative regulatory role in certain biological processes, such as B cell receptor (BCR) signaling pathways, BCR signaling pathways, and immunoglobulin (Ig)-mediated immune responses in BRCA, triglyceride metabolic processes and linoleic acid metabolism in KICH, and immune response activating cell surface receptor signaling pathways in THCA (**Figure 10A**). To delve deeper into the biological role of *RAB5B* in these tumor types, GSVA was conducted. **Figure 10B** showcases the top 15 pathways that exhibit significant positive or negative correlations with *RAB5B* expression. The analysis results showed that the expression of *RAB5B* was significantly associated with a variety of immune-related pathways, including RAGE receptor binding, antifungal humoral response, positive regulation of killing of other organisms’ cells, neuroactive ligand-receptor interaction, primary immunodeficiency, IgE binding, miRNA loading onto RISC involved in miRNA-mediated gene silencing, RISC loading complex, transplant rejection, systemic lupus erythematosus, antigen processing and presentation, graft-versus-host disease, positive regulation of antigen processing and presentation, immune complex clearance, intestinal immune network for IgA production, and primary immunodeficiency. In addition, numerous pathways related to immune factors, such as negative regulation of interleukin-1α production, chemokine signaling pathway, cytokine-cytokine receptor interaction, interleukin-21 production, negative regulation of interleukin-21 production, immunoglobulin receptor activity, chemokine receptor binding, and cytokine and cytokine receptor interaction, were all associated with *RAB5B*.

### 3.9 Study on the relationship between *RAB5B* expression level and drug sensitivity and molecular docking of *RAB5B* targeting compounds

**Figure 11:**
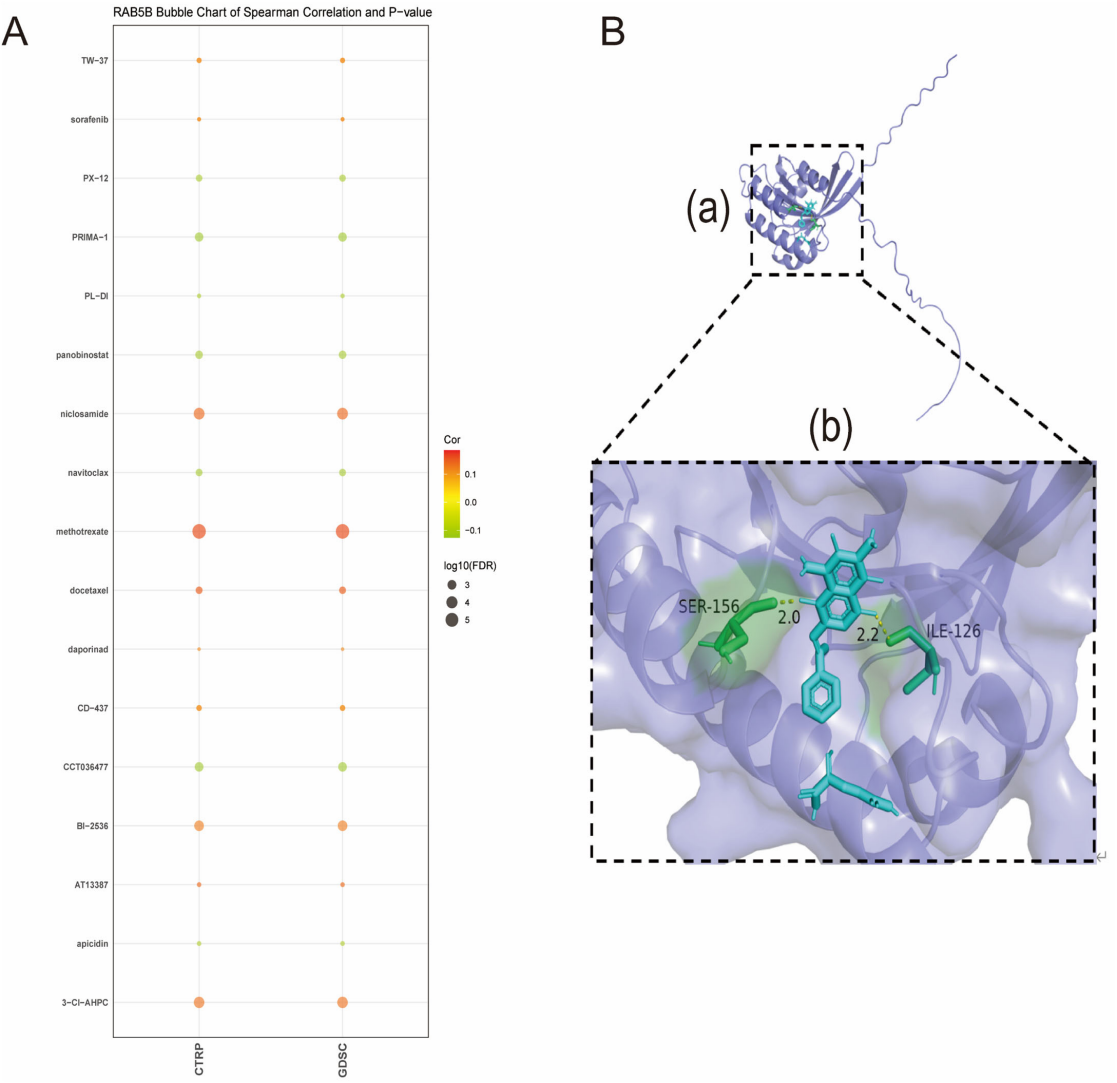
Association of *RAB5B* expression with drug sensitivity and molecular docking of *RAB5B*-targeting compounds. **(A)** The relationships between *RAB5B* expression and predicted drug responses in CTRP and GDSC cancer cell lines were displayed. **(B)** A close-up view of the interaction between methotrexate and the *RAB5B* protein was presented, highlighting the ligand-binding site (a) through structural annotations. Additionally, a magnified perspective of this critical interaction region (b) was provided, where important receptor residues were represented by sticks and labeled, with the receptor displayed in a purplish-green cartoon rendering, and methotrexate (blue) and receptor residues involved in ligand binding (green) were explicitly visualized.

**Figure 11A** reveals the list of drugs with significant correlation with the expression of *RAB5B*, showing that the expression level of *RAB5B* is positively correlated with the response to a series of drugs such as TW-37, sorafenib, niclosamide, methotrexate, docetaxel, daporinad, CD-437, BI-2536, AT13387, and 3-CL-AHPC. This analysis was conducted using data from the Cancer Therapeutics Response Portal (CTRP) and the Genomics of Drug Sensitivity in Cancer (GDSC) datasets, which provided comprehensive information on drug responses and gene expression profiles across a wide range of cancer cell lines.On the contrary, the expression of *RAB5B* was negatively correlated with the response to numerous cancer medications such as PX-12, PRIMA-1, PL-DI, panobinostat, navitoclax, CCT036477 and apicidin. To assess the binding affinity of drugs screened from CTPR and GDSC databases to *RAB5B* proteins, molecular docking analysis was performed for drugs with the most significant affinity using Autodock4 software. This analysis not only reveals the binding sites and interaction details of drugs and proteins, but also calculates the binding energy of each interaction.

Methotrexate, a chemotherapeutic drug, plays a key role in the treatment of various cancers (26), mainly by inhibiting dihydrofolate reductase to block the synthesis of tumor cells, thus inhibiting the growth and proliferation of tumors. Studies have shown that methotrexate, as a first-line chemotherapy drug, has shown remarkable efficacy in the treatment of breast cancer, and its therapeutic effect is closely related to the sensitivity of drugs to breast cancer cells (27). Methotrexate was selected for in-depth molecular docking analysis. The results showed that there was a significant hydrogen bond between methotrexate and *RAB5B* protein, accompanied by strong electrostatic interaction (as shown in **Figure 11B**). The optimized conformation shows the binding energy of 2.23 kJ/mol, which strongly indicates that the binding between them is extremely stable.

### 3.10 Cell proliferation and migration of silencing *RAB5B*

**Figure 12:**
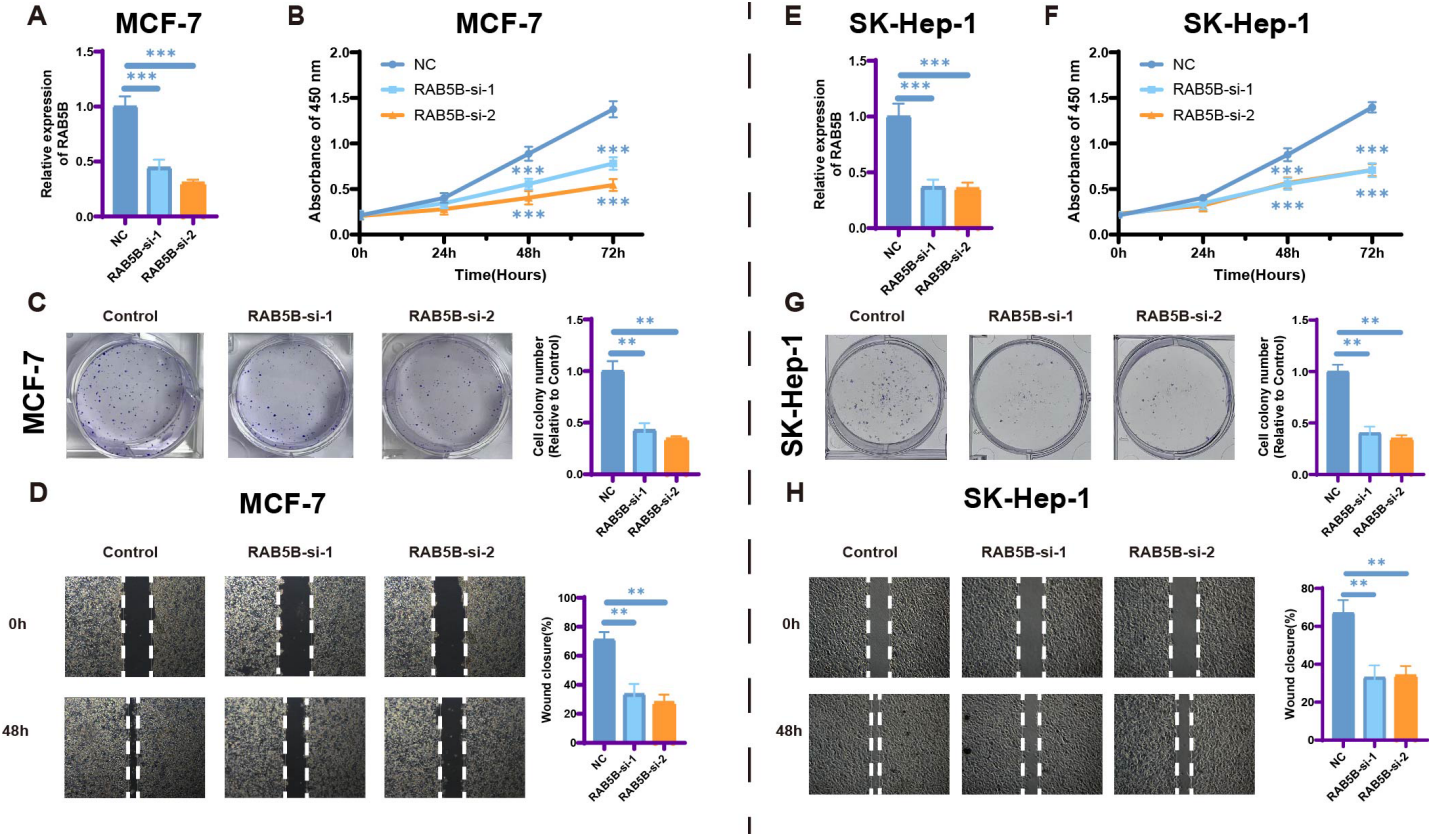
Silencing *RAB5B* leads to inhibition of cell proliferation and migration. **(A)** The relative mRNA expression levels of RAB5B were analyzed and displayed across *RAB5B*-si-1, *RAB5B*-si-2, and NC control groups in the *RAB5B*-knockdown MCF-7 cancer cell line model (Statistical significance was marked as ***p-values < 0.001). **(B)** CCK-8 assays showed that knocking down *RAB5B*. (Statistical significance was marked as ***p-values <0.001). **(C)** Clone formation assays were performed to compare the clonogenic potential between the *RAB5B*-si-1, *RAB5B*-si-2, and control cell lines. (Statistical significance was marked as **p-values < 0.01). **(D)** Wound healing assays were also executed to evaluate the migratory capacity of cancer cells in the *RAB5B*-si-1, *RAB5B*-si-2, and NC groups (mark to **p < 0.01). **(E)** The relative mRNA expression levels of *RAB5B* were analyzed and displayed across *RAB5B*-si-1, *RAB5B*-si-2, and NC control groups in the *RAB5B*-knockdown SK-Hep-1cancer cell line model (Statistical significance was marked as ***p-values < 0.001). **(F)** CCK-8 assays showed that knocking down *RAB5B*. (Statistical significance was marked as ***p-values < 0.001). **(G)** Clone formation assays were performed to compare the clonogenic potential between the *RAB5B*-si-1, *RAB5B*-si-2, and control cell lines. (Statistical significance was marked as **p-values < 0.01). **(H)** Wound healing assays were also executed to evaluate the migratory capacity of cancer cells in the *RAB5B*-si-1, *RAB5B*-si-2, and NC groups (mark to **p < 0.01). In Figure **(A, C, E, G)** The values were normalized to the mean of the control group within each independent biological replicate.

The impact of *RAB5B* silencing on cell proliferation and migration was investigated to better understand its role in cancer progression. Cancer cell lines (MCF-7, SK-Hep-1) with *RAB5B* knockdown via siRNA were employed to explore its biological functions. Results from CCK-8 assays (**Figures 12B, F**) demonstrated significantly reduced proliferation in *RAB5B*-deficient cells compared to control groups, highlighting the importance of *RAB5B* in regulating cancer cell proliferation. Colony formation assays presented in **Figures 12C, G** further supported these findings, showing fewer colonies formed in *RAB5B*-knockdown cells and confirming that *RAB5B* silencing impairs the clonogenic potential of these cells.The effect of *RAB5B* silencing on cell migration was also evaluated using wound healing assays (**Figures 12D, H**). At both 0 hours and 48 hours, *RAB5B*-knockdown cells exhibited significantly reduced migration capacity compared to control cells, as indicated by smaller wound closure areas. This suggests that *RAB5B* plays a critical role in promoting the migratory properties of cancer cells, which is essential for tumor metastasis.The conserved suppression of proliferation and migration across distinct cancer types provides robust evidence for *RAB5B*’s critical role in cancer progression. These findings significantly advance our understanding of *RAB5B* as a pan-cancer oncogene and validate its potential as a therapeutic target, while highlighting the need for further *in vivo* validation and mechanistic dissection.

## 4 Discussion

The blocking of immune checkpoints has brought a major breakthrough for cancer immunotherapy (28), and cancer patients have seen the dawn of cure. However, its efficacy varies across cancers, with only a minority responding well due to differences in tumor microenvironments. Studies have identified *RAB5B* as a robust prognostic biomarker for cancer, capable of predicting immunotherapy outcomes. In addition, our research results also provide useful clues for further exploring the potential role of *RAB5B* in cancer immunotherapy.

The *RAB5B* gene, a key molecule in intracellular vesicle transport, has garnered significant attention in biology and medical research. As a member of the *RAB* family, it regulates early endocytosis by controlling vesicle formation and fusion, crucial for cellular material exchange and signal transduction (29). *RAB5B* interacts with effector proteins to precisely regulate the transport of membrane vesicles from the plasma membrane to early endosomes, crucial for cellular substance exchange and signal transduction. Normally, it helps maintain cellular homeostasis and physiological function (30). However, when the *RAB5B* gene is mutated or expressed abnormally, the cell membrane vesicle transport system may be seriously disturbed (31). In some cancers, the expression level or active state of *RAB5B* may change, which affects the efficiency and accuracy of vesicle transport, and then promotes the proliferation, migration and invasion of tumor cells. In addition, the abnormality of *RAB5B* may interfere with other important signal pathways in cells and further aggravate the malignant progress of tumor. *RAB5B* exhibits varying expression patterns and functions across different cancers. In some, high *RAB5B* expression correlates with tumor malignancy, poor prognosis, and drug resistance, while in others, it may act as a tumor suppressor, with downregulation linked to tumor development. These differences may stem from variations in cancer genetics, microenvironment, and signaling pathways. In addition, *RAB5B* is also involved in the regulation of autophagy, which further broadens its potential role in cancer research. In cancer, the regulatory effect of *RAB5B* on autophagy may directly or indirectly affect the proliferation, apoptosis and drug resistance of tumor cells. As research progresses, the mechanisms of *RAB5B* in cancer will be elucidated, offering new insights and targets for cancer diagnosis, treatment, and prognosis assessment.

To better understand the cellular mechanisms of *RAB5B*, a comprehensive oncology study was carried out using large databases like TCGA, HPA, CCLE, GTEx, and TISCH. The study examined *RAB5B* gene expression fluctuations, patient survival and prognosis, genetic variations, and immune cell infiltration. It focused on genomic variations of *RAB5B* across different cancers and their prognostic impact. Additionally, the study explored potential links between *RAB5B* and factors such as CNV, DNA methylation, MSI, TMB, and immune cell infiltration in the tumor microenvironment. Firstly, data resources from the TCGA and GTEx databases were utilized to conduct a comprehensive and systematic analysis of the expression patterns of *RAB5B* across various cancers. The results showed that the expression of *RAB5B* in KIRC and KICH was down-regulated. However, the expression of *RAB5B* in other eight cancers is abnormally increased, which is quite different from the previous view that the expression of *RAB5B* is inhibited in invasive malignant tumors, thus suggesting that *RAB5B* may play a completely different role in many cancers. Further exploration of the TISCH single-cell database revealed that the expression of *RAB5B* is primarily concentrated in specific cell types within the tumor immune microenvironment, especially endothelial cells and immune cells. The expression level of *RAB5B* is particularly significant in the immune cell subtypes of basal cell carcinoma and melanoma.

Subsequent comprehensive analysis examined cancer patient prognosis across multiple clinical endpoints, including OS, DSS, DFI, and PFS. The results show that there is a significant correlation between the expression level of *RAB5B* and the prognosis of cancer patients. Specifically, the high expression of *RAB5B* in UCEC and THCA is closely related to the poor prognosis of patients, while the expression in ACC, BRCA and CESC is related to the good prognosis. These findings further emphasize the importance of *RAB5B* in predicting the survival and prognosis of cancer patients, and also verify its value as a reliable prognostic marker.

Subsequently, the cBioPortal database was used to investigate the genetic variations of *RAB5B*. The findings highlighted that *RAB5B* amplification was particularly prominent among UCS patients. Consequently, the study focused on *RAB5B* genomic variations, especially its amplification in UCS. Additionally, the correlation between CNV, DNA methylation, and *RAB5B* gene expression was analyzed. The results indicated a positive correlation between *RAB5B* expression levels and both CNV degree and DNA methylation levels in UVM patients. When *RAB5B* copy number is significantly elevated and methylation levels are low, patients often exhibit more severe conditions and poorer prognoses. Therefore, the *RAB5B* gene may serve as an important prognostic marker in UVM, aiding doctors in assessing disease progression and patient survival rates. Furthermore, the abnormal expression of the *RAB5B* gene may primarily be driven by variations in DNA copy number and changes in methylation status, which could be key factors contributing to its dysfunction. This discovery offers a new direction for future research, potentially aiding in the development of therapeutic strategies targeting *RAB5B*-related mechanisms.

Although TMB has become a universal prognostic indicator for cancer patients after immunotherapy (32), which provides an important basis for the choice of immunotherapy schemes in the era of precision medicine, MSI and specific clinicopathological features, such as the increase of TMB and lymphocyte infiltration, are the core prognostic factors in the evaluation of the efficacy of PD-1 inhibitors (33). Pan-cancer analysis reveals notable associations between *RAB5B* expression and TMB levels across 11 malignancies, as well as MSI status in 4 tumor types. These observations prompt speculation that *RAB5B* might participate in modulating TMB/MSI pathways within tumors, potentially influencing therapeutic responses to immune checkpoint blockade. Therefore, future research should focus on influencing tumor microenvironment or MSI by regulating *RAB5B*, in order to fully explore the expression of *RAB5B* in predicting the efficacy of immunotherapy and accurately stratifying the patient population. Through careful analysis of two related cohorts, the internal relationship between the expression of *RAB5B* and the response of immune checkpoint inhibitors (such as anti-PD-L1 and anti-PD-1 antibodies) was deeply explored, and the reliability of *RAB5B* as a potential biomarker for predicting the sensitivity of immune checkpoint blocking therapy was strongly verified. Especially in the field of urinary system tumors, it is confirmed that *RAB5B* is a reliable and effective prognostic marker for predicting the response to anti-PD-1 immunotherapy. However, to comprehensively and accurately assess the practical application value of *RAB5B* as an immunotherapy biomarker, additional cohort studies are necessary for support and verification. Across various tumor types, the expression of MMR genes demonstrates a significant positive correlation with *RAB5B* levels, particularly in ACC, CHOL, KICH, KIRC, KIRP, LIHC, PCPG, PRAD, THCA, and TYHM. *RAB5B* has the potential to decrease TMB and MSI in certain cancers through the MMR system. Consequently, future efforts may enable the effective evaluation of immunotherapy’s actual therapeutic effect by monitoring *RAB5B* expression levels, facilitating the customization of personalized treatment plans for patients and ultimately enhancing therapeutic outcomes. In our research, the key role of *RAB5B* in tumor immunity has been clearly verified. The characteristics of TME are not only an important index to predict the response of tumor cell immunotherapy, but also closely related to the clinical outcome of patients (34). Under normal physiological conditions, the immune system has a strong ability to detect and eliminate tumor cells. However, tumor cells can skillfully avoid the surveillance of the immune system by using various survival and proliferation strategies. Through ESTIMATE analysis, it was observed that the expression level of *RAB5B* was negatively correlated with the matrix score, immune score, and ESTIMATE score in 33 tumor microenvironment cases, further confirming *RAB5B*’s significant role in regulating the tumor microenvironment. In addition, this discovery may also indicate that the high expression of *RAB5B* is closely related to the inhibitory state in tumor microenvironment and the weakening of immune response, which may provide favorable conditions for the escape and further development of tumor cells.

Many studies have emphasized the profound influence of tumor microenvironment, especially mummy microenvironment, on tumor growth, immune escape and treatment resistance (35). Infiltrating immune cells in tumor microenvironment play a vital role, and they have a dual role in the occurrence and progress of tumors (36): they can both promote and inhibit. Against this backdrop, an in-depth investigation was conducted into the relationship between *RAB5B* expression and immune cell infiltration. The findings reveal a substantial correlation between *RAB5B* expression levels and the extent of immune cell infiltration across various cancer types, strongly supported by experimental evidence. Leveraging extensive cancer data from the TCGA database, with a specific focus on ACC and THYM cancers, the study identified a negative correlation between *RAB5B* expression and the infiltration levels of NKT, CD8+T, MDSC, Tfh, and γ/δ T cells. Conversely, *RAB5B* expression was positively correlated with neutrophil infiltration levels in B cells, tumor-associated fibroblasts, endothelial cells, CD4+ T cells, and mast cells. This suggests that *RAB5B* may influence cancer progression and patient prognosis by modulating the tumor microenvironment. Moreover, studies have shown that increased neutrophil infiltration in ACC and THYM is closely linked to short-term tumor recurrence and poor prognosis (37, 38), which is consistent with the results of our prognosis analysis. Therefore, it is hypothesized that in patients with ACC and THYM, *RAB5B* may exert its biological function by modulating the number of M2-type macrophages in the tumor microenvironment, thereby adversely impacting patient prognosis. This hypothesis provides new clues and ideas for us to further explore the specific mechanism of *RAB5B* in the process of tumor immunity.

Our research has brought novel insights into the application of *RAB5B* in oncology, and clearly revealed its close relationship with immune cells and immune-related molecules in various cancer types. Specifically, it was found that the expression of *RAB5B* is positively correlated with a range of immunomodulatory genes, encompassing MHC molecules, immune activating factors, immunosuppressive molecules, chemokines, and their receptor proteins, among others, in almost all cancer types. This discovery not only emphasizes the close relationship between the expression of *RAB5B* and the immune infiltration of tumor cells, but also suggests that it may have an important impact on the prognosis of patients, thus opening up new potential targets for the development of immunomodulatory drugs.

Utilizing the cBioPortal database, it was observed that the mutation frequency of the *RAB5B* gene is elevated in patients with BRCA. Further analysis highlighted a significant correlation between *RAB5B* gene mutations and survival indices in KICH patients. Meanwhile, data from the HPA database indicated that high *RAB5B* expression is associated with a better prognosis in THCA patients, serving as a positive prognostic factor. GSEA results revealed that high *RAB5B* expression is closely linked to immune regulation and stress response, including humoral immune responses mediated by circulating immunoglobulins, cellular responses to unfolded proteins, and autoimmune thyroid diseases. Additionally, *RAB5B* expression was negatively correlated with several immune-related pathways, such as B cell-mediated immunity, immunoglobulin-mediated immune responses, and phagocytosis, which are crucial for tumor cell stress response, repair mechanisms, and cell cycle regulation. This suggests that *RAB5B* may influence the tumor immune environment through these pathways. GSVA further explored the relationship between *RAB5B* and immune-related pathways across different cancer types, offering new insights into *RAB5B*’s role as an immunomodulator. These findings not only elucidate the potential mechanisms of *RAB5B* but also provide valuable clues for developing cancer immunotherapy strategies targeting *RAB5B* in the future.

In subsequent research, a series of potential anticancer drugs were screened to investigate their potential therapeutic role by targeting *RAB5B*.The expression level of *RAB5B* in tumor is used to guide drug selection, and the drug sensitivity analysis of *RAB5B* shows that methotrexate has great potential to develop new molecular targeted therapy. Methotrexate is a chemotherapy drug widely used in many cancers, including acute leukemia, malignant lymphoma, lung cancer, breast cancer and ovarian cancer. It is an anti-folic acid antitumor drug, and its mechanism of action involves many aspects, which together inhibit the growth and proliferation of cancer cells. Mainly by inhibiting dihydrofolate reductase, it can reduce the production of tetrahydrofolate, and then interfere with the synthesis of DNA, RNA and protein (39). This interference not only blocks the normal metabolism and function of cancer cells, but also directly affects the DNA synthesis process. Methotrexate also interferes with the expression of activation-related genes and protein synthesis of T lymphocytes by affecting the metabolic process in cells, such as inhibiting DNA synthesis, thus reducing the activation and proliferation of T lymphocytes and regulating the immune response of the body (40). In addition, the research results show that methotrexate has shown remarkable efficacy in the treatment of breast cancer, which suggests that the treatment strategy for *RAB5B* may provide a new direction for drug development of some cancers. As a chemotherapy drug, methotrexate plays an important role in cancer treatment, and its multi-target mechanism provides more possibilities for cancer treatment.

By knocking down *RAB5B* in MCF-7and SK-Hep-1cells, it is strongly proved that *RAB5B* significantly inhibits the proliferation and migration of breast cancer cells, highlighting its potential as a target for anti-breast cancer drugs. However, this study, which integrates information from multiple databases, faces limitations, particularly in the transcriptomics data analysis method. To enhance the reliability of the conclusions and improve clinical relevance, more in-depth *in vitro* experiments are necessary in the future.

To sum up, the *RAB5B* gene holds irreplaceable importance in pan-cancer research. The study demonstrated that *RAB5B* expression levels have complex and diverse correlations with core elements of various tumor types, including immune regulatory factors, immune cell infiltration, tumor microenvironment characteristics, TMB, and MSI, leading to different effects on tumor immunity. This conclusion was strongly verified through in-depth pan-cancer analysis. *RAB5B* is crucial not only for maintaining normal cellular transcription but also for cancer initiation, progression, and deterioration. As scientific research advances, there is substantial evidence to suggest that thorough exploration of the *RAB5B* gene and its associated pathways may offer novel approaches and strategies for cancer diagnosis, treatment, and prevention.

## Data availability

The datasets presented in this article are not readily available because None. Requests to access the datasets should be directed to jiangchao1212@126.com
