# Supplementary material for: RAB5B: explore its carcinogenic potential and understand its role, prognostic value and biological significance in pan-cancer
Source: Front Immunol. 2025 Aug 6;16:1619733. doi: 10.3389/fimmu.2025.1619733 (PMC12364618; doi:10.3389/fimmu.2025.1619733)
Supplement: Supplementary file 1 [file Table1.docx]

**SUPPLEMENTAL MATERIALS**

**SUPPLEMENTAL TABLE**

**TABLE S1 TCGA cancer abbreviations and the corresponding cancer type**

| Abbreviations | Cancer Type |
| --- | --- |
| ACC | Adrenocortical carcinoma |
| BLCA | Bladder Urothelial Carcinoma |
| BRCA | Breast invasive carcinoma |
| CESC | Cervical squamous cell carcinoma and endocervical adenocarcinoma |
| CHOL | Cholangiocarcinoma |
| COAD | Colon adenocarcinoma |
| DLBC | Lymphoid Neoplasm Diffuse Large B- cell Lymphoma |
| ESCA | Esophageal carcinoma |
| GBM | Glioblastoma multiforme |
| HNSC | Head and Neck squamous cell carcinoma |
| KICH | Kidney Chromophobe |
| KIRC | Kidney renal clear cell carcinoma |
| KIRP | Kidney renal papillary cell carcinoma |
| LAML | Acute Myeloid Leukemia |
| LGG | Brain Lower Grade Glioma |
| LIHC | Liver hepatocellular carcinoma |
| LUAD | Lung adenocarcinoma |
| LUSC | Lung squamous cell carcinoma |
| MESO | Mesothelioma |
| OV | Ovarian serous cystadenocarcinoma |
| PAAD | Pancreatic adenocarcinoma |
| PCPG | Pheochromocytoma and Paraganglioma |
| PRAD | Prostate adenocarcinoma |
| READ | Rectum adenocarcinoma |
| SARC | Sarcoma |
| SKCM | Skin Cutaneous Melanoma |
| STAD | Stomach adenocarcinoma |
| TGCT | Testicular Germ Cell Tumors |
| THCA | Thyroid carcinoma |
| THYM | Thymoma |
| UCEC | Uterine Corpus Endometrial Carcinoma |
| UCS | Uterine Carcinosarcoma |
| UVM | Uveal Melanoma |

**Table S2.** **siRNA sequences of NC and RAB5B used in our experiments**

|  | sense（5'-3'） | antisense（5'-3'） |
| --- | --- | --- |
| si-NC | UUCUCCGAACGUGUCACGUTT | ACGUGACACGUUCGGAGAATT |
| si- RAB5B #1 | CAGAGGAUUGGUGGAUGAGUGCACA | UGUGCACUCAUCCACCAAUCCUCUG |
| si- RAB5B #2 | GGAUUGGUGGAUGAGUGCACAUAUA | UAUAUGUGCACUCAUCCACCAAUCC |

**Table S3.** **Primer sequence of β-actin and RAB5B used in our experiments**

| Gene name | Forward primer，5’-3’ | Reverse primer，3’-5’ |
| --- | --- | --- |
| β-actin (Human) | GGCATCCTCACCCTGAAGTACC | CCACACGCAGCTCATTGTAGAAG |
| RAB5B(Human) | AAATTGGTCCTGCTGGGAGAATCTG | CTGGTACTCATGGAACTGCCCTTTG |
